# Structure and epitope distribution of heparan sulfate is disrupted in experimental lung hypoplasia: a glycobiological epigenetic cause for malformation?

**DOI:** 10.1186/1471-213X-11-38

**Published:** 2011-06-14

**Authors:** Sophie M Thompson, Marilyn G Connell, Toin H van Kuppevelt, Ruoyan Xu, Jeremy E Turnbull, Paul D Losty, David G Fernig, Edwin C Jesudason

**Affiliations:** 1 Institute of Integrative Biology, University of Liverpool, Crown Street, Liverpool, UK; 2 Division of Child Health, School of Reproductive and Developmental Medicine, Royal Liverpool Children's Hospital, Alder Hey, Liverpool, UK; 3 Department of Biochemistry 280, Nijmegen Centre for Molecular Life Sciences, Radboud University Nijmegen Medical Centre, P.O. Box 9101, 6500 HB Nijmegen, The Netherlands

## Abstract

**Background:**

Heparan sulfate (HS) is present on the surface of virtually all mammalian cells and is a major component of the extracellular matrix (ECM), where it plays a pivotal role in cell-cell and cell-matrix cross-talk through its large interactome. Disruption of HS biosynthesis in mice results in neonatal death as a consequence of malformed lungs, indicating that HS is crucial for airway morphogenesis. Neonatal mortality (~50%) in newborns with congenital diaphragmatic hernia (CDH) is principally associated with lung hypoplasia and pulmonary hypertension. Given the importance of HS for lung morphogenesis, we investigated developmental changes in HS structure in normal and hypoplastic lungs using the nitrofen rat model of CDH and semi-synthetic bacteriophage ('phage) display antibodies, which identify distinct HS structures.

**Results:**

The pulmonary pattern of elaborated HS structures is developmentally regulated. For example, the HS4E4V epitope is highly expressed in sub-epithelial mesenchyme of E15.5 - E17.5 lungs and at a lower level in more distal mesenchyme. However, by E19.5, this epitope is expressed similarly throughout the lung mesenchyme.

We also reveal abnormalities in HS fine structure and spatiotemporal distribution of HS epitopes in hypoplastic CDH lungs. These changes involve structures recognised by key growth factors, FGF2 and FGF9. For example, the EV3C3V epitope, which was abnormally distributed in the mesenchyme of hypoplastic lungs, is recognised by FGF2.

**Conclusions:**

The observed spatiotemporal changes in HS structure during normal lung development will likely reflect altered activities of many HS-binding proteins regulating lung morphogenesis. Abnormalities in HS structure and distribution in hypoplastic lungs can be expected to perturb HS:protein interactions, ECM microenvironments and crucial epithelial-mesenchyme communication, which may contribute to lung dysmorphogenesis. Indeed, a number of epitopes correlate with structures recognised by FGFs, suggesting a functional consequence of the observed changes in HS in these lungs. These results identify a novel, significant molecular defect in hypoplastic lungs and reveals HS as a potential contributor to hypoplastic lung development in CDH. Finally, these results afford the prospect that HS-mimetic therapeutics could repair defective signalling in hypoplastic lungs, improve lung growth, and reduce CDH mortality.

## Background

The majority of the extracellular proteins involved in regulating embryonic development interact with heparin/heparan sulfate (HS), and, moreover, require HS for their cellular activities [1]. These include proteins required for lung morphogenesis [2,3]. For example, not only are fibroblast growth factors (FGFs) essential for lung development [4-10], but they require HS for FGF receptor activation and subsequent signalling [11-13]. Due to its vast interactome and location at the cell surface and within the extracellular matrix (ECM), HS is ideally positioned to integrate biochemical regulators of lung development with mechanical stimuli required for normal lung growth [14,15].

HS is a linear polysaccharide consisting of N-acetyl glucosamine-glucuronic acid disaccharide repeats. Chains are variably modified by N-deacetylation/N-sulfation of N-acetyl glucosamines, O-sulfation at various positions and conversion of glucuronic acid to its C-5 epimer, iduronic acid. These modifications do not occur at every potential site within a chain, resulting in a diverse range of HS chain structures displayed by a cell [1]. Moreover, HS is post-synthetically remodelled by 6-O-endosulfatase enzymes, which selectively remove sulphate groups [16-18]. HS chains are usually attached to core proteins to form HS proteoglycans (HSPGs), which are expressed by most mammalian cells and represent a major component of the cell surface and ECM. Individual cells of a tissue display a variety of HS chains, which, in addition to being structurally complex and diverse, are dynamic, altering over time and with cellular physiology [3]. Since interactions between HS and proteins are mediated by specific HS structures, changes in HS structure *in vivo *are likely to alter HS:protein binding events and related signalling. Characterising HS fine structure *in vivo *is, therefore, important as it equates to a view of HS function.

Obtaining structural information on native HS is challenging due to the non-template nature of HS biosynthesis (unlike proteins or nucleic acids). Tissue HS is typically analysed by extraction and purification. However, the inherent averaging of this approach limits the information to an overall assessment of the mixed population of HS structures present, and all spatial information is lost. In addition, due to the relative low immunogenicity of HS, only a limited number of HS specific monoclonal antibodies are available [19-21]. However, single chain variable fragment (scFv) antibodies generated by bacteriophage ('phage) display methodology [22,23] allow specific classes of structures in HS to be probed *in situ*. We have demonstrated recently that these antibodies display distinct specificities for different HS structures *in vitro *and, therefore, the individual HS epitopes they recognise, though structurally complex, are unique [24]. Limited analysis of one antibody shows that these probes are suitable to identify the diversity of HS in different cellular compartments of fetal lungs [25].

HS plays a fundamental role in airway morphogenesis. In *Drosophila *and mice, disrupted HS biosynthesis *in vivo *results in defective airway branching, which in the mouse, results in lethal neonatal respiratory insufficiency [26-29]. In addition, digestion of endogenous HS in cultured lung explants using heparitinases or inhibition of HS sulfation with sodium chlorate, disrupts branching [30,31]. Further compelling evidence for a critical role of HS in lung disease is the association in humans and mice of mutations in the HSPG, glypican-3, with lung hypoplasia and congenital diaphragmatic hernia (CDH), known as Simpson-Golabi-Behmel syndrome [32-35]. It is, therefore, clear that HS is crucial for lung development and identifies HS as a potential contributor to pulmonary pathologies such as pulmonary hypoplasia in CDH [3].

CDH is characterised by a diaphragmatic defect, herniation of abdominal contents into the thoracic cavity and pulmonary hypoplasia. The high neonatal morbidity and mortality is largely attributed to severe respiratory insufficiency resulting from hypoplastic lung growth and pulmonary hypertension. Hitherto, most work exploring the pathogenesis of human birth defects has focussed on the identification of changes in gene expression and/or in protein levels. However, very few truly genetic causes of these defects have been identified. Since strong evidence supports a central role for HS as a master regulator of extracellular proteins controlling embryogenesis, we have investigated HS structure during development of the lung, and in the pathogenesis of CDH and pulmonary hypoplasia using HS specific 'phage display antibodies. We utilised the teratogen-induced rodent model of CDH, which uses nitrofen (2,4-dichlorophenyl-*p*-nitrophenyl ether) to induce congenital malformations in the offspring of treated pregnant dams with striking similarity to human CDH [36-38]. Here, we demonstrate that HS undergoes structural alterations during normal lung development and that there are pronounced abnormalities of HS structure and epitope distribution in hypoplastic lungs. Aberrations in epithelial basement membrane structure and composition were also identified in hypoplastic lungs, reflecting a specific abnormality in the ECM. The functional importance of these changes to HS structure during normal development and in hypoplastic lung is illustrated by our finding that a number of the HS epitopes are analogous to structures recognised by the critical morphogenetic growth factors, FGF2 and FGF9. Hence, these novel glycobiological defects may contribute to defective lung morphogenesis via altered interactions between HS and key signalling molecules such as FGFs. In addition, altered contacts between the ECM and cell surface are likely to interrupt mechanotransduction across a tissue, which is crucial for morphogenesis. HS may, therefore, play significant biochemical and biomechanical roles in the pathogenesis of pulmonary hypoplasia in CDH.

## Results

Seven HS 'phage display antibodies were chosen on the basis that they have been raised against HS/heparin from a variety of tissue sources and display distinct binding specificities, which we have characterised in depth [24]. The data on antibody binding specificities and epitope structures are summarised in Table 1.

**Table 1:** Binding specificities of HS antibodies and characteristics of the recognised epitopes, extracted from [24]

Antibody	Sulfation preference	Disulfated preference	Binds monosulfated?	Oligosaccharide length required for significant binding
HS3B7V	Hep > 2S,NS > 6S,NS > HS > 6S,2S > 6S > 2S	2S, NS or 6S, NS	2S or 6S	>dp10

HS4E4V	HS > 6S,NS = 2S,NS > Hep > 6S > 6S,2S	2S, NS or 6S, NS	Only 6S	dp8

HS3A8V	Hep > 2S,NS = HS > 6S,NS > 6S > 6S,2S	2S, NS	Only 6S	dp6

AO4B08V	Hep > HS > 2S,NS > 6S > NS,6S > 6S,2S > 2S	2S, NS	2S or 6S	dp4

EV3C3V	Hep > 2S,NS > HS > 6S,NS > 6S,2S ≥ 6S	2S, NS	Only 6S	dp8

EW4G1V	Hep > 2S,NS > HS > 6S,NS ≥ 6S,2S = 6S > 2S	2S, NS	2S or 6S	dp8

(dp) degree of polymerisation, i.e., a disaccharide is a dp2, tetrasaccharide a dp4, *etc*. (Hep) heparin, (HS) heparan sulfate, (NS) N-sulfate, (2S) 2-O-sulfate, (6S) 6-O-sulfate,

### HS structure changes during normal rat lung development

**Figure 1:**
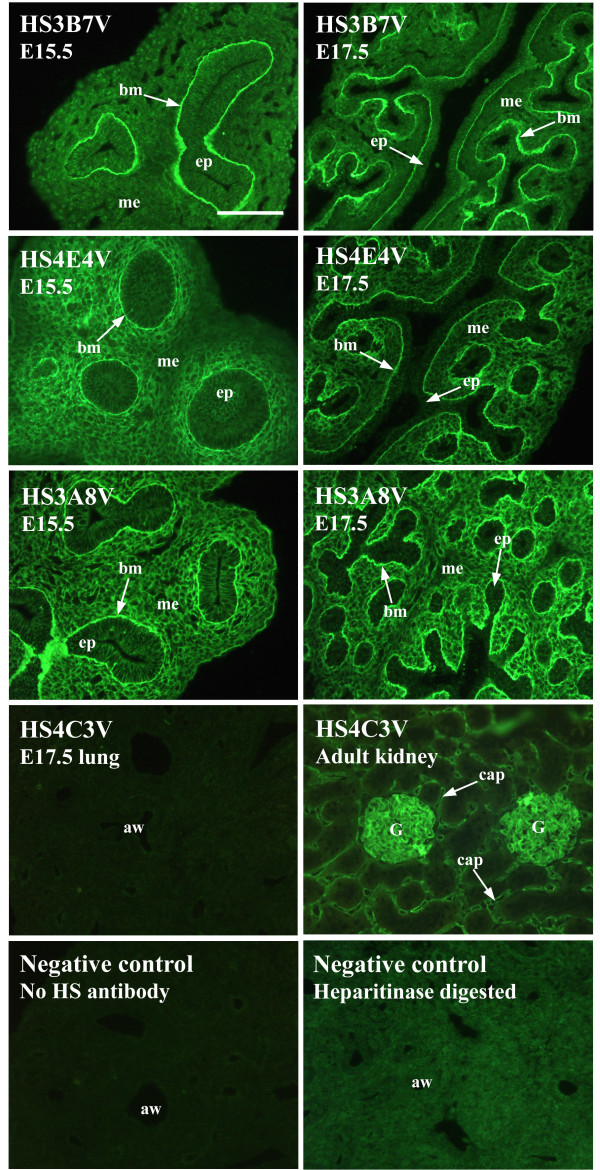
**HS 'phage display antibodies identify distinct epitopes *in situ***. In fetal rat lungs, HS antibodies display different patterns of staining. HS3B7V exclusively labels epithelial basement membranes, whereas HS4E4V and HS3A8V show a more widespread staining pattern. In addition to epithelial basement membrane staining, HS4E4V labels sub-epithelial mesenchymal cells surrounding smaller distal airways and HS3A8V highlights the entire lung mesenchyme and in addition, stains epithelial cells at E15.5. One antibody, HS4C3V, did not stain fetal rat lungs of any developmental age; however, positive staining of adult rat kidney confirmed the functionality of HS4C3V in immunohistochemistry. E15.5 and E17.5 rat lungs and adult rat kidney were probed with HS antibodies followed by rabbit VSV-G tag antibody and FITC conjugated goat anti-rabbit IgG. Negative controls were omission of HS antibody or digestion of HS with heparitinase prior to antibody incubation (HS4E4V shown, heparitinase digest controls for other antibodies are shown in additional files). Scale bar represents 10 μm and all images are the same magnification. (ep) epithelium, (me) mesenchyme, (bm) basement membrane, (aw) airway, (G) glomerulus, (cap) peritubular capillary.

Different staining patterns were observed in developing rat lung with the various HS antibodies, indicating recognition of distinct HS epitope structures (Figure 1 and Table 2).

**Table 2:** Summary of the spatiotemporal distribution of HS epitopes in normal and nitrofen-treated hypoplastic fetal lungs

	E13.5		E15.5		E17.5		E19.5		E21.5	
	Cont	Nitr	Cont	Nitr	Cont	Nitr	Cont	Nitr	Cont	Nitr
**HS3B7V**										

Epithelium	-	n/a	-	-	-	-	-	-	-	-
BM	-	n/a	+++	++	+++	++	+++	++	++	+
Mesenchyme;										
Sub-epithelial	-	n/a	-	-	-	-	-	+	+	+
Sub-mesothelial	-	n/a	-	-	-	-	-	-	+	+

**HS4E4V**										

Epithelium	-	n/a	-	-	-	-	-	-	-	-
BM	+++	n/a	+++	+	+++	++	+++	++	++	++
Mesenchyme;										
Sub-epithelial	+	n/a	+++	+/-	++	+	++	++	++	++
Sub-mesothelial	-	n/a	+	-	+	-	++	++	++	++

**HS3A8V**										

Epithelium	-	n/a	++	-	+	-	-	-	-	-
BM	+++	n/a	+++	++	+++	++	+++	++	+++	++
Mesenchyme;										
Sub-epithelial	-	n/a	+++	++	+++	++	++	++	++	++
Sub-mesothelial	-	n/a	++	++	+	++	++	++	++	++

**AO4B08V**										

Epithelium	-	n/a	++	+	+	-	-	-	-	-
BM	+	n/a	+++	+	+++	+	+++	-	++	+
Mesenchyme;										
Sub-epithelial	-	n/a	-	+	++	++	++	++	++	++
Sub-mesothelial	-	n/a	-	+	+	++	++	++	++	++

**EV3C3V**										

Epithelium	++	n/a	++	-	+	-	-	-	-	-
BM	+++	n/a	+++	++	+++	++	+++	++	+++	++
Mesenchyme;										
Sub-epithelial	+	n/a	+++	++	+++	+++	+++	+++	++	++
Sub-mesothelial	+	n/a	+	++	+	+++	+++	+++	++	++

**EW4G1V**										

Epithelium	-	n/a	+	-	-	-	-	-	-	-
BM	-	n/a	+++	+	+++	++	+++	++	+++	++
Mesenchyme;										
Sub-epithelial	-	n/a	+	+	+	+	++	+++	++	++
Sub-mesothelial	-	n/a	-	-	-	-	++	++	++	++

Levels of immunostaining in the various lung compartments were scored blind by two independent observers with images that were representative of three separate lung samples, as follows: -

- no staining, +/- very low staining, + low staining, ++ moderate staining, +++ high staining

**Figure 2:**
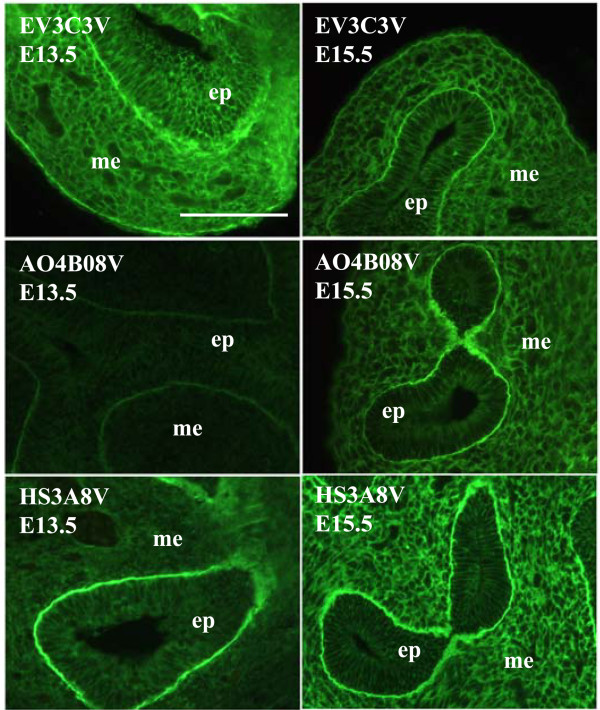
**Different compartments of the lung display distinct HS epitopes e.g., airway epithelium**. Airway epithelial cells display HS epitopes identified by EV3C3V, AO4B08V and HS3A8V. At E13.5, only the EV3C3V epitope is present and at E15.5, AO4B08V and HS3A8V epitopes are additionally displayed. HS structures recognised by HS3B7V, HS4E4V and EW4G1V are not identified in the epithelium at any developmental stage. E13.5 and E15.5 rat lungs were probed with HS antibodies followed by rabbit VSV-G tag antibody and FITC conjugated goat anti-rabbit IgG. Scale bar represents 10 μm and all images are the same magnification. (ep) epithelium, (me) mesenchyme.

**Figure 3:**
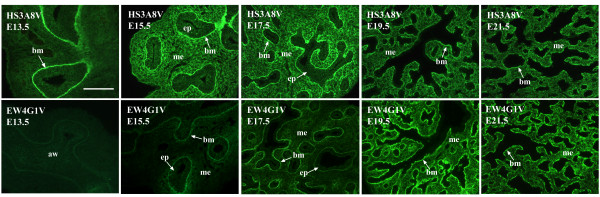
**The spatial distribution of HS epitopes changes during mammalian lung morphogenesis**. Antibody epitopes change in their pattern of distribution during the course of lung development. For example, the epitope recognised by HS3A8V is displayed exclusively by the epithelial basement membranes at E13.5, however, by E15.5, it is also present in the mesenchyme and on the surface of epithelial cells. At E17.5, the epitope is lost from the epithelium, and sub-epithelial mesenchymal expression is briefly increased, before becoming more widespread at E19.5 - E21.5. In contrast, the HS epitope recognised by EW4G1V is not present in E13.5 rat lungs and is only weakly expressed at E15.5 in epithelial basement membranes and mesenchyme surrounding smaller distal airways. Levels of this epitope increase considerably in epithelial basement membranes at E17.5, while mesenchymal expression remains relatively low and concentrated in sub-epithelial areas until E19.5 - E21.5, where epitope distribution becomes more widespread. E13.5 - E21.3 rat lungs were probed with HS antibodies followed by rabbit VSV-G tag antibody and FITC conjugated goat anti-rabbit IgG. Scale bar represents 10 μm and all images are the same magnification. (aw) airway, (bm) basement membrane, (me) mesenchyme, (ep) epithelium.

One particular HS epitope recognised by HS4C3V was not identified in developing rat lungs of any age. The use of adult rat kidney as a positive control demonstrates that this structure is indeed absent from lung (Figure 1). The remaining six antibodies show distinct patterns of staining in fetal rat lungs, and the various lung compartments display different HS structures (additional files 1, 2, 3, 4, 5 and 6, summarised in Table 2). For example, in airway epithelium, HS structures recognised by EV3C3V, AO4B08V and HS3A8V are displayed, whereas the other three antibody epitopes are not. At E13.5, only the EV3C3V HS epitope is displayed by the epithelium, and at E15.5, the AO4B08V and HS3A8V HS epitopes are also present (Figure 2). The spatial localisation of these HS epitopes was shown to change during lung morphogenesis, indicating alterations in the structure of native HS chains during mammalian lung development (Figures 1 and 3 and additional files 1, 2, 3, 4, 5 and 6). For example, the HS epitope recognised by HS3A8V is displayed exclusively by epithelial basement membranes at E13.5 at a high level. At the early pseudoglandular period (E15.5), this structure is additionally displayed at a high level throughout the mesenchyme and by the airway epithelium. At the late pseudoglandular period (E17.5), mesenchymal expression of this epitope remains high; however, distribution of the structure becomes particularly concentrated around smaller developing airways and epithelial staining is no longer present. At canalicular (E19.5) and saccular (E21.5) fetal stages, HS3A8V epitope levels in the mesenchyme decrease and epitope distribution becomes more widespread (Figure 3 and Table 2). In contrast, the HS epitope identified by EW4G1V is not present in E13.5 rat lungs; however, at E15.5 it is detected in epithelial basement membranes and in the surrounding mesenchyme at a low level (Figure 3 and Table 2). At E17.5, levels of this HS epitope drastically increase, particularly in epithelial basement membranes, while epitope levels in the mesenchyme remain low and its distribution becomes concentrated around smaller distal airways. From E19.5 - E21.5, distribution of the EW4G1V epitope becomes more widespread throughout the mesenchyme (Figure 3 and Table 2).

**Figure 4:**
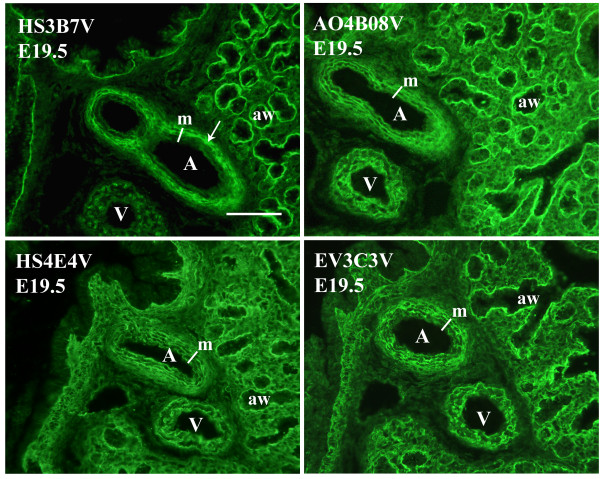
**HS epitope distribution in pulmonary vasculature**. HS3B7V shows a unique pattern of vascular staining in fetal lungs, specifically highlighting the outer tunica media of arterial walls (arrow) and leaving the inner tunica media unlabelled. In addition, this antibody does not stain pulmonary veins. (Some weak, nuclear staining was observed with HS3B7V on occasion, including in veins. However, this was not sensitive to heparinase digestion and is therefore non-specific staining). The remaining antibodies highlight the entire tunica media layer in the walls of both arteries and veins (only AO4B08V, HS4E4V and EV3C3V are shown, however, HS3A8V and HS4E4V display comparable blood vessel staining). Fetal rat lungs were probed with HS antibodies followed by rabbit VSV-G tag antibody and FITC conjugated goat anti-rabbit IgG. Scale bar represents 10 μm and all images are the same magnification. (A) artery, (V) vein, (aw) airway, (m) media.

In the pulmonary vasculature, HS3B7V shows a unique staining profile, specifically highlighting regions in the outer tunica media of arterial walls (Figure 4). This HS structure is absent from the inner medial layer, in contrast to the other five antibody epitopes, which were present and showed comparable vascular staining, highlighting the medial layer of both arteries and veins, predominantly the basal lamina surrounding smooth muscle cell layers (Figure 4).

### HS structure is abnormal in hypoplastic lungs from nitrofen-induced CDH

**Figure 5:**
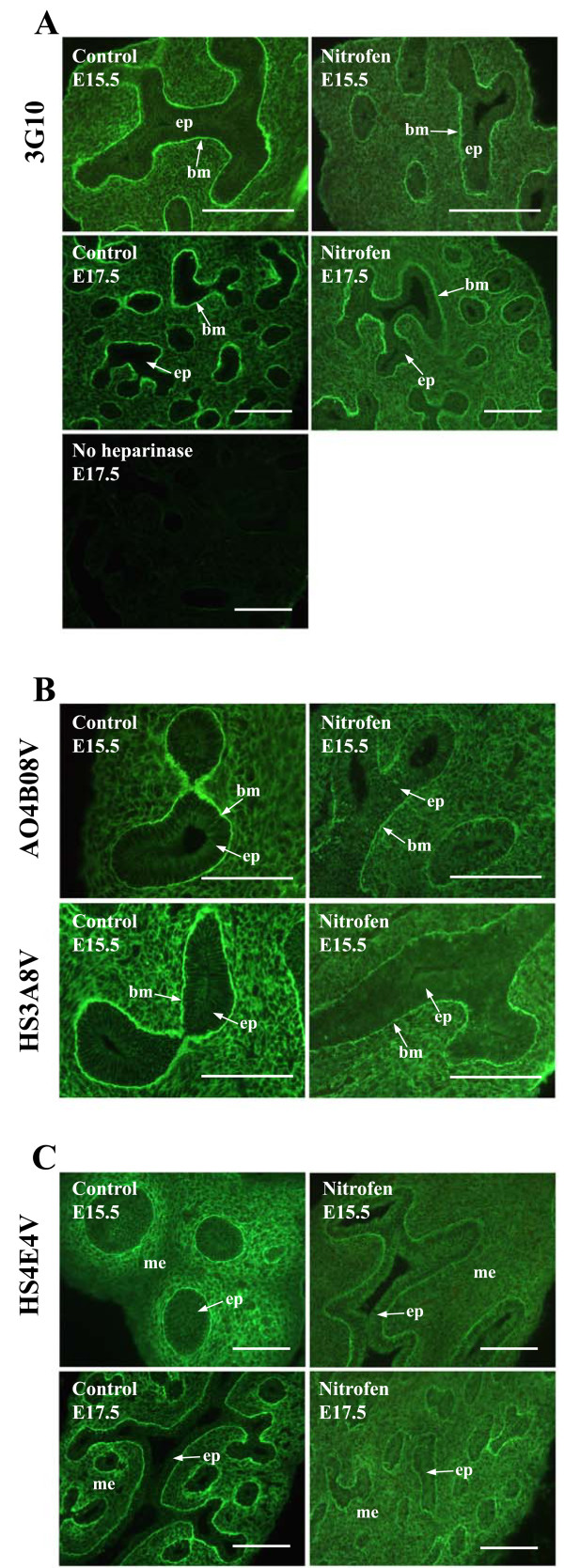
**HS structure is abnormal in hypoplastic nitrofen treated rat lungs**. HSPG levels, identified by 3G10, are reduced in hypoplastic rat lungs, particularly at E15.5 and E17.5 and in epithelial basement membranes (A). Analysis of specific HS epitopes with 'phage display antibodies revealed an abnormality in HS fine structure. A number of epitopes are reduced or lost from the epithelium e.g., AO4B08V and HS3A8V, respectively (B). In addition, a number of epitopes, e.g., HS4E4V, are reduced in the lung mesenchyme (C) and all epitopes are reduced in epithelial basement membranes (B, C). Hypoplastic lungs from rats with nitrofen-induced left sided CDH and control lungs from rats fed olive oil alone were probed with 3G10 after initial digestion of lung HS with heparitinase to reveal the 3G10 neo-epitope on all HSPGs. Bound antibody was then detected with FITC conjugated goat anti-mouse IgG. As a negative control, sections were incubated with heparitinase buffer alone without enzyme, leaving the 3G10 neo-epitope concealed. Incubation of lung sections with HS 'phage display antibodies was followed by rabbit VSV-G tag antibody and FITC conjugated goat anti-rabbit IgG. Scale bars represent 10 μm. (ep) epithelium, (bm) basement membrane, (me) mesenchyme.

**Figure 6:**
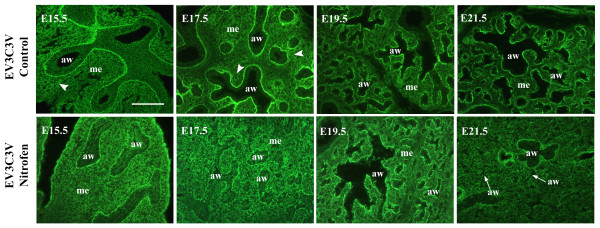
**Spatiotemporal distribution of HS epitopes is abnormal in hypoplastic lungs**. In normal development, a gradient of EV3C3V epitope distribution is observed in the mesenchyme of E15.5 - E17.5 lungs, with high epitope levels in sub-epithelial mesenchyme around distal airways (arrowhead) and lower levels around proximal airways. This organised gradient of EV3C3V epitope distribution is lost in lungs of nitrofen treated rats, which display the structure at a high level throughout the mesenchyme at E15.5 - E17.5. At E19.5 - E21.5, EV3C3V epitope levels and distribution are comparable to control lungs. Hypoplastic lungs from rats with nitrofen-induced left sided CDH and control lungs from rats fed olive oil alone were probed with EV3C3V followed by rabbit VSV-G tag antibody and FITC conjugated goat anti-rabbit IgG. Scale bar represents 10 μm and all images are the same magnification. (aw) airway, (me) mesenchyme.

**Figure 7:**
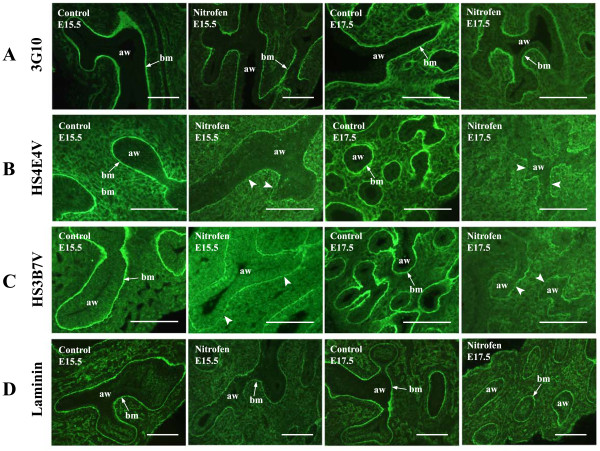
**Airway epithelial basement membranes are abnormal in hypoplastic lungs**. Epithelial basement membranes appear thinner in nitrofen treated lungs, with reduced levels of HSPGs, identified by 3G10 antibody (A) and HS epitopes identified by 'phage display HS antibodies, e.g., HS4E4V and HS3B7V (B, C). Discontinuities in basement membrane HS staining were also observed with HS antibody staining (B, C, arrowheads). This was not apparent with 3G10 immunohistochemistry, identifying all HSPGs (A). To visualise the general structure of basement membranes and assess whether the observed abnormalities are HS specific or a general defect in basement membrane structure, lungs were probed with an antibody to laminin (D). Staining with anti-laminin revealed thinner basement membranes, however, no discontinuities were observed. Hypoplastic lungs from rats with nitrofen-induced left sided CDH and control lungs from rats fed olive oil alone were probed with HS antibodies, 3G10 (after digestion of endogenous HS with heparitinase to reveal the 3G10 neo-epitope on all HSPGs) or anti-laminin antibody. Bound HS antibodies were detected with rabbit VSV-G tag antibody followed by FITC conjugated goat anti-rabbit IgG, 3G10 was detected with FITC conjugated goat anti-mouse IgG and anti-laminin was detected with FITC conjugated goat anti-rabbit IgG. Scale bars represent 10 μm. (aw) airway, (bm) basement membrane, (me) mesenchyme, (ep) epithelium.

Identification of HSPGs with the 3G10 antibody, which recognises the neo-epitope generated on all HSPGs following heparitinase digestion of the HS chains, indicated that there is no gross disruption in the overall spatial localisation of HSPGs in hypoplastic lungs. However, levels of HSPGs are reduced, particularly at E15.5 - E17.5 and in epithelial basement membranes (Figure 5A). Analysis of specific HS epitopes with 'phage display antibodies indicated an abnormality in the fine structure of HS in hypoplastic lungs, which was also more marked in lungs of earlier gestation (Figures 5, 6 and 7, summarised in Table 2). Levels of a number of HS epitopes are reduced (AO4B08V) or lost (HS3A8V, EV3C3V and EW4G1V) from the airway epithelium at E15.5 and E17.5 compared to normal lungs (Figure 5B and Table 2). Moreover, all of the HS structures analysed are displayed at a lower level by epithelial basement membranes. Although a number of HS structures are also shown to be reduced in hypoplastic lung mesenchyme (HS4E4V, HS3A8V and AO4B08V at E15.5 - E17.5) (Figure 5C and additional files 2, 3 and 4), HS structural alterations are more complex than a simple reduction or loss of epitopes. Levels of the HS epitope identified by EV3C3V are increased in hypoplastic lung mesenchyme, and in addition, the spatial distribution of this HS structure is abnormal (Figure 6 and Table 2). In E15.5 - E17.5 control lung mesenchyme, a gradient of EV3C3V epitope distribution is observed, with sub-epithelial areas adjacent to smaller, distal airways displaying high epitope levels and more proximal regions of the lung displaying a lower level of the structure. However, in nitrofen treated hypoplastic lungs, this gradient is not present and epitope distribution is more widespread throughout the entire lung mesenchyme (Figure 6 and Table 2). At E19.5 - E21.5, EV3C3V epitope levels and distribution are comparable to that observed in control lungs.

Pulmonary arteries of nitrofen-treated hypoplastic lungs have thickened vessel walls with increased smooth muscle content, contributing to the persistent pulmonary hypertension associated with CDH [39-41]. Although we confirmed marked thickening in arterial walls of nitrofen-treated lungs, no difference in HS or HSPG staining was observed. All HS antibodies highlighted the tunica media, with five predominantly staining the basal lamina surrounding the layers of smooth muscle and HS3B7V specifically highlighting an outer region of the arterial walls.

### HS/HSPG staining identifies abnormalities in epithelial basement membranes

Abnormalities in epithelial basement membrane HSPG expression and HS structure were identified in hypoplastic lungs. Basement membranes appear thinner, and levels of both HSPGs (Figure 7A) and specific HS epitopes (Figure 7B and 7C) are reduced. In addition, staining with HS antibodies revealed discontinuities in basement membrane HS distribution, which are not observed with 3G10 immunohistochemistry.

To examine the general structure of epithelial basement membranes in hypoplastic lungs and evaluate whether abnormalities are HS/HSPG specific, hypoplastic rat lungs were probed with an antibody to laminin (Figure 7D), an integral component of basement membranes. Immunohistochemical detection of laminin indicated that epithelial basement membranes are indeed thinner; however, no discontinuities in laminin staining were observed (Figure 7D). The abnormally fine laminin staining of epithelial basement membranes was more pronounced in lungs at the pseudoglandular stages of development (E15.5 and E17.5), reminiscent of HS and HSPG basement membrane staining.

### Functional analysis of HS epitopes using ELISA

**Figure 8:**
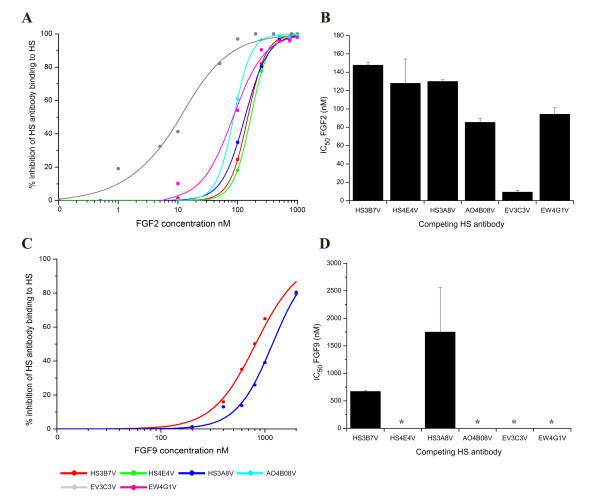
**Functional analysis of antibody epitope structures via competition ELISA with FGF2 and FGF9**. FGF2 and FGF9 competed with a number of antibodies for HS binding, indicating that epitope structures are analogous to structures recognised by these growth factors. FGF2 competed for all six epitopes to variable extents, but most significantly with EV3C3V. FGF9, in contrast, showed more competitive selectivity and was only able to compete for two epitope structures, recognised by HS3B7V and HS3A8V. PMHS was biotinylated and immobilised on streptavidin coated microtitre plates. Equilibrium binding of HS antibodies in the presence of various concentrations of FGF2 (A, B) or FGF9 (C, D) were quantified at *A*490 using an anti-VSV-G tag antibody (P5D4) followed by HRP conjugated anti-mouse antibody and OPD substrate. Absorbance values were converted to % inhibition of antibody binding to HS by FGFs and plotted against FGF concentration (A, C). Curves were generated using OriginPro and a non-linear logistic dose response fit. IC_50 _values (B, D) were calculated as the concentration of FGF required for 50% inhibition of antibody binding to immobilised HS. Values are the mean of triplicate samples, *error bars *represent the S.E and the data are representative of two separate experiments. * denotes antibody epitopes not competed for by FGF.

To further our understanding of the functional consequences of abnormal HS structure identified in hypoplastic lungs, we analysed the specificities of the HS antibodies in competition ELISAs with FGF2 and FGF9, which are known to be involved in lung morphogenesis [4,42-44]. The relative binding affinities of FGFs for HS epitopes were evaluated by determining IC_50 _values, defined as the concentration of FGF that inhibits antibody binding to HS by 50% (Figure 8). This allowed us to investigate possible overlap between antibody epitope structures and FGF binding sites in HS, to identify potential biological functions of the antibody epitopes.

FGF2 competed for all six antibody epitopes to varying extents (Figure 8A and 8B), whereas FGF9 only competed for two antibody epitopes, recognised by HS3B7V and HS3A8V (Figure 8C and 8D). The ability of FGF2 to compete for HS3B7V, HS4E4V and HS3A8V epitopes was similar, with IC_50 _values of 150 nM ± 2.8 nM, 130 nM ± 26 nM and 130 nM ± 1.9 nM. FGF2 also showed comparable competition for AO4B08V and EW4G1V epitopes, with IC_50 _values of 85 nM ± 4.2 nM and 94 nM ± 7.1 nM. Significant competition for the EV3C3V epitope structure in HS was achieved with FGF2, with an IC_50 _value of 9.3 nM ± 2 nM. In contrast to FGF2, FGF9 only competed with two antibodies, HS3B7V and HS3A8V, for HS binding, with IC_50 _values of 670 nM ± 18 nM and 1.8 μM ± 0.81 μm, respectively (Figure 8C and 8D).

## Discussion and Conclusions

HS is a master regulator of morphogenesis and is essential for lung development. In the present study, we have demonstrated spatiotemporal alterations in HS structure and distribution during normal and hypoplastic lung development using HS specific 'phage display antibodies. Moreover, we show that a number of antibody epitopes are also structures recognised by FGFs, suggesting that abnormal distribution of these epitopes in hypoplastic CDH lungs may alter FGF binding, with functional consequences for morphogenesis.

### HS structure in normal fetal rat lungs

A number of HS scFv antibodies have been used previously to analyse HS structures in adult human lungs [45]. Only one of these antibodies used in the previous work is used in the present study (EV3C3V). In adult lungs, the EV3C3V HS epitope is identified in airway epithelial cells and their underlying basement membranes and in basement membranes surrounding smooth muscle cells of blood vessels [45]. We also identified the EV3C3V epitope in epithelial and smooth muscle cell basement membranes at all developmental stages (Figures 2, 4, 6 and additional file 5) and in airway epithelial cells at E13.5 - E17.5 (Figure 2). This demonstrates a degree of conservation in HS structure between species.

We demonstrate that distinct cellular compartments of the lung display a variety of HS chains of different structure, which are modified during lung development, with temporal variation in both expression level and spatial localisation of HS epitopes identified by HS antibodies.

The epitope for one antibody, HS4C3V, was not identified in fetal lungs, however, it was present in kidney tissue (Figure 1) and has previously been detected in immunoblots of solubilised fetal lung extracts [24]. This raises the possibility of cryptic binding sites for the antibodies *in situ*, and is an important consideration when interpreting immunohistochemical data, i.e., the absence of an epitope *in situ *does not necessarily mean it is not present in a tissue, rather, the epitope may be masked, for example, by endogenous proteins bound to HS. The antibodies, therefore, specifically highlight free binding structures in HS. Indeed, occupied, cryptic binding sites in HS *in situ *have been identified previously in growing mammary glands probed with FGF2 [46].

The antibody binding specificities (Table 1) suggests the nature of the observed changes in HS structure that occur in developing lungs. For example, the HS4E4V HS epitope is not found in E13.5 lungs, and at E15.5 - E17.5, the epitope is identified in the mesenchyme surrounding distal, but not proximal airways (Figure 1 and additional file 2). This can be explained by contrasting patterns of sulfation, e.g., highly sulfated HS modified with both 6-O- and 2-O-sulfates are located in the mesenchyme surrounding proximal airways, while less sulfated HS, modified by 6-O- or 2-O- sulfates is found in mesenchyme surrounding distal airways, where it offers optimal binding for HS4E4V [24]. In contrast to HS4E4V, HS3A8V binds to more highly sulfated HS, particularly N-sulfated sequences (Table 1), and displays a wider epitope distribution throughout fetal lung mesenchyme (Figures 1 and 3). Hence, more highly sulfated HS in proximal mesenchyme may allow binding of HS3A8V, but not HS4E4V, while a more heterogeneous population of HS structures in distal mesenchyme, allows binding of both.

The localisation of specific HS structures within the developing lung and their importance for the coordination of branching morphogenesis has been hinted at in previous studies, e.g., expression of the epitope recognised by the HS specific monoclonal antibody, 10E4, was shown to alter rapidly with lung growth *in vitro *[31]. In addition, blanket addition of heparin to lung cultures lacking endogenous sulfated GAGs after sodium chlorate treatment results in generalised epithelial expansion, indicating a global growth response, rather than defined branching [31]. These data, together with ours, suggest that distinct HS structures are specifically displayed by the various lung cell types to direct localised signalling and spatiotemporally restricted morphogenetic cues, e.g., epithelial budding. In addition, modification of HS fine structure during development, together with alterations in HS microenvironment via dynamic HS:protein interactions, will influence crucial cell-cell and cell-matrix communication governing lung morphogenesis. HS structural dynamics are, therefore, likely to be an important regulator of fetal lung morphogenesis.

### HS structure and epitope distribution are abnormal in hypoplastic CDH lungs

Using the nitrofen rat model of CDH, we investigated the potential role of HS in hypoplastic lung development, since HS has been shown to be crucial for normal lung morphogenesis. In addition, it has been shown previously that nitrofen-exposed rat lung explants respond abnormally to exogenous FGF1 and FGF2 and also heparin, suggesting a possible defect in the FGF:FGFR:HS signalling system [47,48]. Notably, in humans, a mutation in the gene encoding the HSPG, glypican-3, features multiple congenital anomalies, including CDH [32-35].

Following analysis of HS fine structure and its developmental regulation during normal rat lung morphogenesis, HS structure and distribution was analysed in hypoplastic lungs from rats with nitrofen-induced CDH. In hypoplastic lungs, HSPG expression is reduced, and in addition, specific abnormalities in HS structure and spatial distribution were observed, which cannot simply be a consequence of an overall reduction in the level of HSPGs, since the level of some epitopes is increased, e.g., EV3C3V (Figure 6). As the overall distribution of HSPGs appears the same in control and hypoplastic lungs, abnormalities in the spatial distribution of specific epitopes is likely to reflect alterations in HS fine structure and irregular localisation of discrete HS structures displayed by the various lung cell types. In addition, the occupancy or availability of antibody binding sites may differ in hypoplastic lungs due to differences in protein binding events. It is of note that the molecular abnormalities highlighted in hypoplastic lungs are most striking at the pseudoglandular period of respiratory morphogenesis, when the lung is actively branching (E15.5 - E17.5) (Figures 5 and 6 and additional files 1, 2, 3, 4, 5 and 6). This supports previous work, suggesting that in CDH, lungs are intrinsically abnormal from early stages of organogenesis and that lung defects emerge in tandem alongside the hernia [49-51].

Abnormal HS structure has been shown to result in defective lung development in previous studies, e.g., in mice lacking HS biosynthetic enzymes, NDST-1 or C5-epimerase [27,28]. In *Drosophila*, RNAi of HS 6-O-sulfotransferase (HS6ST) perturbs FGF signalling and disrupts primary branching of the tracheal system [52] and, similarly, a proportion of mutant *Drosophila *embryos lacking functional HS6ST develop tracheal branching defects [53]. The generation of double mutants lacking functional 2-O-sulfotransferase (HS2ST) and HS6ST is completely lethal, with disrupted FGF signalling and a failure of tracheal precursor cells to migrate to form primary branches [53]. In the mammalian lung, chemical inhibition of HS sulfation prevents FGF10 induced epithelial budding [30] and HS6ST1 deficient mice exhibit impaired alveolarisation [54]. In contrast to previous studies where HS biosynthesis has been perturbed and effects on the lung sought, the present study investigates an established developmental malformation (pulmonary hypoplasia, CDH) to show for the first time that hypoplastic lungs feature substantial abnormalities of HS structure and epitope distribution. Moreover, previous studies in which HS has been disrupted, e.g., by deletion of genes encoding HS biosynthetic enzymes, have not characterised in any depth the actual effects on HS structure and epitope distribution in the malformed lung.

Changes in HS structure in the lung is likely to modify HS:protein interactions, since these rely on specific HS structures [1]. This in turn, will affect various signalling systems, as HS:protein interactions have been shown to be functionally significant, regulating transport and effector functions of the protein ligand. For example, HS plays a key role in FGF signalling, which is fundamental for lung morphogenesis.

HS facilitates interactions between FGFs and FGFRs and is also required for sustained FGFR activation and subsequent cellular signalling [11-13]. Cells expressing FGFRs but lacking HSPGs are unresponsive to FGF unless heparin/HS is added [11,12] and treatment of cells with sodium chlorate or heparinase blocks the biological activity of FGFs, an effect which can be restored by the addition of exogenous heparin [13]. In an *ex vivo *model system of epithelial branching morphogenesis using mouse salivary gland, modification of FGF:HS binding affinities was shown to impact upon the morphogenetic effect of FGFs [55]. FGF10 with a reduced affinity for HS (via single amino acid mutations in the heparin binding site), formed abnormal gradients due to altered transport properties, resulting in an induction of epithelial branching rather than elongation observed with wild type FGF10 [55]. In contrast, reduced affinity of FGF10 for its receptor, FGFR2b, affected only the extent of the response, without altering the nature of the response.

Abnormal HS structure and distribution observed here in hypoplastic lungs can, therefore, be expected to contribute to defective lung morphogenesis via aberrant epithelial-mesenchymal signalling as a result of altered HS:protein interactions.

### Competitive selectivity of FGFs and antibodies provides insight into structure:function relationships of epitopes and a functional consequence of abnormal HS in hypoplastic lungs

Competitive binding assays with FGFs and HS antibodies allowed us to demonstrate that antibodies recognise specific HS structures that are also recognised by key FGF morphogens, thereby revealing biological relevance of epitopes. Alterations in FGF bindings sites in the lung has previously been shown to have functional consequences for morphogenesis [30].

FGF2 is expressed in the developing lung and is important for lung morphogenesis [43,44,56]. The HS binding specificity of FGF2 is well characterised, requiring N-sulfated and 2-O-sulfated HS structures [57,58] and at least a tetrasaccharide [59] for binding. In competitive binding assays, FGF2 competed with all six HS antibodies to varying extents. Of particular note is the effectiveness of FGF2 to compete with EV3C3V, which was significantly higher compared to competition with the other antibodies (Figure 8A and 8B). Previous characterisation of epitope structures reveals that EV3C3V binding structures are analogous to those recognised by FGF2. Both require N-sulfation and 2-O-sulfation for binding, with little sensitivity to the presence or absence of 6-O-sulfates (Table 1) [24,57]. The other epitopes appear to overlap in part with structures recognised by FGF2, requiring a higher concentration of FGF2 to successfully compete with the antibodies. These structures may represent lower affinity binding sites for FGF2, which have been previously identified in HS [60].

Similarly, FGF9 is essential for normal lung development [4,42,61,62]. In binding assays, FGF9 showed more selective competition than FGF2, competing only for the epitopes recognised by HS3B7V and HS3A8V (Figure 8C and 8D). Although the binding specificity of FGF9 is not as well characterised as that of FGF2, the competitive binding data for FGF9 illustrates the biological relevance of the observed variation in these epitopes in normal and hypoplastic prenatal lungs. FGF9 competes most effectively for HS3B7V epitopes, with an IC_50 _value almost three times lower than with HS3A8V (670 nM compared to 1.8 μM). We have previously shown that HS3B7V requires longer HS structures for significant binding (Table 1) [24], and this may also reflect the binding specificity of FGF9. As FGF9 has been shown to readily form homodimers in solution [63,64], the requirement for longer HS structures for binding may not be that surprising.

Functional analysis of epitope structures enables us to suggest potential biological consequences of abnormal epitope distribution in developing lungs. In CDH hypoplastic lungs, the EV3C3V epitope was identified at a higher level compared to normal lungs, and in addition, was shown to be abnormally distributed in hypoplastic lung mesenchyme (Figure 6). This may indicate an increase in the number of EV3C3V epitopes and, therefore, structures recognised by FGF2, in the HS synthesised by the lung cells. Alternatively, the availability of these structures may be increased in the lung mesenchyme due to altered expression of proteins that bind this class of structures. Moreover, these lungs respond abnormally to FGF2 [47]. Addition of FGF2 to nitrofen-treated lung explants results in increased lung area and formation of dilated, cystic airways, whereas FGF2 was shown to have minimal effect on the growth of control lung explants [47]. Our data describing an increased number of available EV3C3V/FGF2 binding structures in the mesenchyme of hypoplastic lungs provides a possible explanation for this abnormal response to exogenous FGF2.

Epitopes analogous to structures recognised by FGF9, i.e., the HS3A8V and HS3B7V epitopes, were also abnormally expressed in nitrofen-treated lungs. Both epitopes were expressed at a reduced level in epithelial basement membranes and in addition, showed abnormal mesenchymal expression (Table 2 and additional files 1 and 3). The HS3B7V epitope was displayed at a low level in sub-epithelial mesenchymal compartments of E19.5 hypoplastic lungs, and this is a compartment which was not stained in normal lungs (Table 2 and additional file 1). The HS3A8V epitope was identified at a high level in sub-epithelial mesenchyme at E15.5 and E17.5 in normal lungs, however, in hypoplastic lungs, mesenchymal expression was reduced and the partitioning of HS3A8V staining in sub-epithelial and sub-mesothelial mesenchyme was not evident (Table 2 and additional file 3). Thus, these HS structures recognised by FGF9 are either synthesised at an abnormal frequency in HS chains, or the availability of these structures in HS chains is reduced in an irregular manner. FGF9 has been shown to be particularly important for mesenchymal growth and differentiation [4,42,62,65] and mesenchymal-epithelial signalling through regulation of other morphogen expression levels [62]. An abnormal distribution of HS structures able to bind FGF9 in the lung mesenchyme may, therefore, have a significant effect on FGF9 transport between cellular compartments and/or activity, including downstream effects on other morphogens regulated by FGF9, for example, SHH and FGF7 and FGF10 [42,62]. Collectively, these data suggest that observed changes in HS structure in hypoplastic lungs is likely to result in altered interactions with critical regulatory proteins, such as FGFs, leading to irregular cell signalling and epithelial-mesenchymal cross-talk, which may ultimately contribute to defective lung morphogenesis.

### Abnormal HS may help explain disrupted mechanobiology in hypoplastic lungs

The ECM facilitates communication between cellular and extracellular environments, including mechanotransduction [66-68]. Prenatal airway smooth muscle (ASM) exhibits periodic contractility resulting in rhythmic airway peristalsis, which moves lung liquid along the airways and induces a distending pressure in end buds, stretching the lung to promote growth [69-72]. Demonstrating a specific link between HS dependent signalling and airway contractility, FGF10 is synthesised by these ASM cells [73] and stimulates airway peristalsis [70]. However, in hypoplastic lungs from the nitrofen CDH model, reduced FGF10 levels [74] accompany abnormal airway peristalsis [75,76]. Given the large HS interactome, the structural changes in HS identified here provide a promising possible explanation for the mechanical abnormalities measured in hypoplastic lungs.

### Epithelial basement membranes are abnormal in hypoplastic lungs

Basement membranes are specialised extracellular matrices underlying epithelial and endothelial cells [77] composed mainly of collagen IV, laminin, nidogen/entactin and proteoglycans including HSPGs, perlecan [78], agrin [79,80] and collagen XVIII [81,82]. HSPGs contribute to basement membrane assembly, structure and function: e.g., when grown in sodium chlorate, murine teratocarcinoma cells develop an incorrectly assembled basement membrane [83]. In addition, heparin and HS influence associations between basement membrane components, laminin and collagen IV [84,85]. HSPGs also regulate growth factor activity by controlling their transport through the matrix and sequestering them to form local reservoirs [86-89]. In the developing lung, basement membranes are pivotal for epithelial-mesenchymal cross talk and irregular assembly and structure of airway basement membranes is detrimental to lung morphogenesis [90,91].

We have shown here that hypoplastic lungs exhibit abnormal epithelial basement membranes. Immunodetection of laminin, HSPGs and HS structures demonstrated an abnormally thinned basement membrane. Additionally, HS epitopes, but not HSPGs or laminin, were displayed discontinuously in hypoplastic lungs, suggesting abnormal localisation of HS epitopes and/or availability of binding sites in HS.

### Clinical implications

Current treatments for CDH primarily focus on postnatal management to address the consequences of pulmonary hypoplasia and hypertension, including strategies aimed at providing adequate tissue oxygenation via inhaled nitric oxide, high frequency oscillatory ventilation and extracorporeal membrane oxygenation (ECMO). However, CDH mortality has not been greatly improved. Results from the present work suggest that heparin/HS based therapeutics may be beneficial in ameliorating hypoplastic lung growth in CDH. Indeed, a number of glycotherapeutics are emerging for the repair of damaged tissue, e.g., skin and bone [92-96] and for the treatment of some cancers [97-99]. The morphogenetic effect of chemically modified heparins has previously been investigated in salivary gland branching morphogenesis [100]. Using a similar rationale, investigating the effect of various engineered heparins on lung growth may help develop a class of HS structures able to ameliorate lung hypoplasia, reduce smooth muscle cell proliferation and increase vascular branching. With chemically modified heparins available, which possess low or zero anticoagulant activity [101], this is an exciting potential future therapeutic avenue.

## Methods

### Lung retrieval and induction of CDH

Timed-pregnant Sprague-Dawley rats (Charles River, UK) were gavage fed 100 mg nitrofen (2,4-dichloro-4'-nitrodiphenylether) (Zheijang Chemicals, Hangzhou, China) dissolved in olive oil on day 9.5 of gestation (vaginal plug positive, day 0) to induce left-sided CDH and pulmonary hypoplasia in newborn pups [49]. Control animals received olive oil. Embryos and fetuses were harvested on embryonic day (E)13.5 (controls only), E15.5, E17.5, E19.5 and E21.5 of gestation by caesarean section under terminal anaesthesia (intraperitoneal sodium pentobarbitone). Lungs were dissected out and fixed in 4% (w/v) paraformaldehyde in phosphate buffered saline (PBS) (7.5 mM Na_2_HPO_4_, 2.8 mM NaH_2_PO_4_, 150 mM NaCl pH 7.4). Lungs were washed in PBS, cryoprotected with 20% (w/v) sucrose in PBS overnight and gelatine embedded. Gelatine blocks containing lung tissue were covered in Cryo-M-Bed (Bright, Huntington, UK) and snap frozen in cooled isopentane. Adult kidney was prepared in the same way. Tissue sections were cut at 8 μm on a cryostat, mounted onto chrome alum gel slides and stored at -40°C.

All animal procedures complied with the UK Animal (Scientific Procedures) Act 1986 and were conducted with UK Home Office approval, ref. PPL40/2293.

### Immunohistochemistry

Slides were removed from - 40°C, allowed to thaw and rinsed in PBS. Sections were blocked in 10% (v/v) goat serum in PBS for 2 h at room temperature for all antibodies. Following immunohistochemistry, sections were mounted with fluorescent mounting medium. Scoring of staining was performed blind by two independent observers with images that were representative of at least three separate lung samples.

### HS 'phage display scFv antibodies

HS antibodies were diluted 1/5 in 1% (v/v) goat serum in PBS and incubated with lung sections overnight at 4°C. Bound antibody was detected with rabbit VSV-G tag antibody (Abcam, Cambridge, UK), diluted 1/200 in 1% (v/v) goat serum in PBS, for 2 h at room temperature, followed by FITC conjugated goat anti-rabbit IgG (Sigma-Aldrich, Gillingham, UK), diluted 1/500 in the dark for 1 h. Controls were the omission of HS antibody or treatment of sections with heparitinase (EC 4.2.2.8) (IBEX Technologies Inc, Canada) overnight at 37°C (changing enzyme after 4 h), prior to antibody incubation, to remove HS epitopes.

### HSPG specific antibody, 3G10

In order to reveal the 3G10 neo-epitope in tissue sections, endogenous HS was digested with heparitinase (EC 4.2.2.8) overnight at 37°C, replacing enzyme after 4 h. 3G10 antibody (Seikagaku/AMS Biotechnology, Oxon, UK) was diluted 1/200 in 1% (v/v) goat serum in PBS and incubated with lung sections overnight at 4°C. Bound antibody was detected using FITC conjugated goat anti-mouse IgG (Sigma-Aldrich, Gillingham, UK), incubated in the dark for 1 h. Controls were omission of 3G10 antibody or omission of heparitinase digestion to leave HS chains intact and the 3G10 neo-epitope unavailable.

### Laminin antibody

Rabbit anti-laminin (Sigma-Aldrich, Gillingham, UK) was diluted 1/100 in 1% (v/v) goat serum in PBS and sections incubated overnight at 4°C. Bound antibody was detected with FITC conjugated goat anti-rabbit IgG, diluted 1/200 and incubated in the dark for 1 h.

### FGF2 and FGF9 synthesis and purification

Full-length human recombinant FGF2 with an N-terminal hexahistidine tag was produced in *E. coli *exactly as described [102]. FGF9 (Uniprot Accession: P31371; residues: 1-208) with a 6 × Histidine tag and a TEV cleavage site (26 amino acids, MKHHHHHHPMSDYDIPTTENLYFQGA) at the N-terminus was expressed in C41 E.coli cells using a modified pET-24b vector (pETM-11, kind gift from Dr Paul Elliott, University of Liverpool), which provides the sequences of the 6 × Histidine tag and the TEV cleavage site. Protein was produced in bacteria using an auto induction system [103]. Cells were grown at 37°C, for 7 h in Terrific Broth, and FGF-9 production was induced at 22°C for 16 h. Cell pellets were lysed by sonication in Buffer A9 (0.3 M NaCl, 50 mM Tris, 1 mM DTT, pH7.4) with 0.2 mg/ml lysozyme (lysozyme, chicken egg white, Calbiochem, Nottingham, UK), 10 μg/ml DNase I (deoxyribonuclease I, from bovine pancreas, Sigma-Aldrich, Gillingham, UK), 1 tablet of protease inhibitor (complete EDTA-Free, Protease Inhibitor Cocktail Tablets, Roche, West Sussex, UK), 0.2% (v/v) Tween-20 and 5% (v/v) glycerol. The lysate was loaded onto 5 ml heparin agarose column (Affi-Gel Heparin Gel, Bio-rad, Hemel Hempstead, UK), which was washed with Buffer A9 until the absorbance returned to baseline. FGF-9 was eluted in Buffer B9 (50 mM Tris, 2 M NaCl, 1 mM DTT, pH 7.5). The eluate was diluted 4-fold with 50 mM Tris and applied to a 1 mL HiTrap column (GE Healthcare UK Ltd, Buckinghamshire, UK), and eluted with an imidazole gradeint (50 mM to 500 mM imidazole) in 0.5 M NaCl, 1 mM DTT, 50 mM Tris, pH7.5. Eluted protein was dialysed against 10 mM phosphate, pH 7.5, 1 mM DTT and then stored at -80°C.

### ELISA

Maxisorp 96-well microtitre plates were coated with 3 μg/ml streptavidin (Pierce Biotechnology, IL, USA) in 0.1 M Na_2_CO_3_/0.1 M NaHCO_3 _(pH 9.6) for 16 h at 4°C and then blocked with 1% (w/v) BSA in PBS with 0.05% (v/v) Tween-20 (PBST). Porcine mucosal HS (PMHS) was internally biotinylated with EZ-link NHS-LC-biotin (Pierce Biotechnology, IL, USA) as described previously [24] and plates coated with 100 μg/ml biotinylated PMHS for 2 h at room temperature and then washed with PBST. Fifteen μl of HS antibody was added to each well with 15 μl competitor FGF and incubated overnight at 4°C. Antibody dilutions were determined in a titre and were used at final concentrations of; HS3B7V 1/5, HS4E4V 1/2, HS3A8V 1/20, AO4B08V 1/5, EV3C3V 1/10 and EW4G1V 1/2. FGF2 (1.2 mg/ml) and FGF9 (2.2 mg/ml) in sodium phosphate buffer, pH 7.4 were diluted in 1% (w/v) BSA in PBST to the required concentrations. For a positive control, HS antibodies were added to wells alone, without FGF competitor and for a negative control, HS antibodies were added to wells lacking biotinylated HS. Plates were washed in PBST and bound HS antibody was detected with mouse anti-VSV-G IgG (clone P5D4) (Abcam, Cambridge, UK) diluted 1/2000 in 1% (w/v) BSA in PBST followed by HRP-conjugated sheep anti-mouse IgG (GE Healthcare UK Ltd, Buckinghamshire, UK) diluted 1/2000 in 1% (w/v) BSA in PBST. After a final wash in PBST, plates were developed with o-phenylenediamine (0.8 mg/mL) (AbD Serotec, Oxford, UK) containing 0.03% (v/v) hydrogen peroxide. The reaction was stopped with 0.5 M H_2_SO_4 _and absorbance read at 492 nm.

## Authors' contributions

SMT carried out all the experimental work and wrote the manuscript. MGC carried out animal dissections and assisted in experimental work. RX synthesised and purified FGF2 and FGF9 proteins. THvK provided the phage display antibodies, gave advice on their application and contributed to experimental design of the study. JET, ECJ, DGF and PDL conceived of the study, participated in its design and coordination, obtained funding and edited the manuscript. All authors read and approved the final manuscript.

## Supplementary Material

Additional file 1 **HS3B7V**. Immunohistochemical staining of E13.5 - E21.5 normal lungs and E15.5 - E21.5 hypoplastic lungs with HS3B7V. The HS3B7V HS epitope is localised to the epithelial basement membrane in both control and hypoplastic lungs from E15.5. However, in hypoplastic lungs, expression of this HS structure is reduced and staining of epithelial basement membranes is irregular. In addition, in nitrofen E19.5 lungs, there is additional weak staining identified in sub-epithelial mesenchyme. As a negative control, endogenous HS was digested with heparitinase prior to antibody incubation. (aw) airway, (mes) mesenchyme, (ep) epithelium, (bm) basement membrane, (br) bronchus. Click here for file

Additional file 2 **HS4E4V**. Immunohistochemical staining of E13.5 - E21.5 normal lungs and E15.5 - E21.5 hypoplastic lungs with HS4E4V. In normal lungs, the HS4E4V HS epitope is present in epithelial basement membranes and the surrounding mesenchyme, particularly in sub-epithelial areas adjacent to distal airways. In hypoplastic lungs, expression of this epitope is severely reduced, particularly in epithelial basement membranes and mesenchyme of E15.5 and E17.5 lungs. As a negative control, endogenous HS was digested with heparitinase prior to antibody incubation. (aw) airway, (oe) oesophagus, (mes) mesenchyme, (ep) epithelium, (bm) basement membrane, (br) bronchus. Click here for file

Additional file 3 **HS3A8V**. Immunohistochemical staining of E13.5 - E21.5 normal lungs and E15.5 - E21.5 hypoplastic lungs with HS3A8V. In normal lungs, the HS epitope recognised by HS3A8V is restricted to epithelial basement membranes at E13.5. From E15.5, distribution of the epitope is more widespread and is present in epithelial basement membranes and throughout the mesenchyme, particularly in sub-epithelial mesenchyme. Epithelial cells also display this HS structure transiently at E15.5 and (more weakly) at E17.5. In hypoplastic lungs, mesenchymal expression of the HS3A8V epitope is reduced, particularly at E15.5 and E17.5, and epithelial staining observed in normal lungs is lost. Additionally, irregularities in epithelial basement membrane staining are observed. As a negative control, endogenous HS was digested with heparitinase prior to antibody incubation. (aw) airway, (mes) mesenchyme, (ep) epithelium, (bm) basement membrane, (br) bronchus. Click here for file

Additional file 4 **AO4B08V**. Immunohistochemical staining of E13.5 - E21.5 normal lungs and E15.5 - E21.5 hypoplastic lungs with AO4B08V. Expression of the AO4B08V HS epitope increases during the course of normal lung development. At E13.5, it is only weakly expressed by epithelial basement membranes, and at E15.5, is additionally displayed at a low level in the mesenchyme and airway epithelium. From E17.5 - E21.5, levels of this epitope increases in basement membranes and throughout the mesenchyme. In hypoplastic lungs, however, expression of the AO4B08V epitope is reduced in the epithelium and underlying basement membranes, and in addition, basement membranes appear discontinuous. In lung mesenchyme, however, the AO4B08V epitope structure is displayed at a higher level compared to normal lungs. As a negative control, endogenous HS was digested with heparitinase prior to antibody incubation. (aw) airway, (mes) mesenchyme, (ep) epithelium, (bm) basement membrane, (br) bronchus. Click here for file

Additional file 5 **EV3C3V**. Immunohistochemical staining of E13.5 - E21.5 normal lungs and E15.5 - E21.5 hypoplastic lungs with EV3C3V. In normal lungs, the EV3C3V epitope is displayed by the epithelium at E13.5 - E17.5 and in the underlying basement membranes at E13.5 - E21.5. A gradient of epitope expression is observed in the mesenchyme, with highest levels in sub-epithelial mesenchyme around smaller, distal airways and lower levels in sub-mesothelial mesenchyme. However, in hypoplastic lungs, this gradient of mesenchymal expression is lost, and the EV3C3V epitope is more extensively and evenly distributed throughout the entire mesenchyme. In addition, epithelial staining is lost from hypoplastic lungs and basement membrane staining is irregular. As a negative control, endogenous HS was digested with heparitinase prior to antibody incubation. (aw) airway, (mes) mesenchyme, (ep) epithelium, (bm) basement membrane, (br) bronchus. Click here for file

Additional file 6 **EW4G1V**. Immunohistochemical staining of E13.5 - E21.5 normal lungs and E15.5 - E21.5 hypoplastic lungs with EW4G1V. In normal developing lungs, the HS structure identified by EW4G1V is absent at E13.5. From E15.5 onwards, however, it is present in all epithelial basement membranes and also at a low level in the mesenchyme, with increased levels at E21.5. This epitope is transiently expressed by the epithelium at E15.5. In hypoplastic lungs, levels of this epitope appear to be raised somewhat in the mesenchyme compared to normal lungs and simultaneously reduced in epithelial basement membranes. As a negative control, endogenous HS was digested with heparitinase prior to antibody incubation. (aw) airway, (mes) mesenchyme, (ep) epithelium, (bm) basement membrane, (br) bronchus. Click here for file

## References

[B1] Ori A Wilkinson MC Fernig DG The heparanome and regulation of cell function: structures, functions and challenges Front Biosci 2008 13 4309 4338 18508513 10.2741/3007

[B2] Warburton D Schwarz M Tefft D Flores-Delgado G Anderson KD Cardoso WV The molecular basis of lung morphogenesis Mech Dev 2000 92 55 81 10.1016/S0925-4773(99)00325-1 10704888

[B3] Thompson SM Jesudason EC Turnbull JE Fernig DG Heparan sulfate in lung morphogenesis: The elephant in the room Birth Defects Res C Embryo Today 2010 90 32 44 10.1002/bdrc.20169 20301217

[B4] Colvin JS White AC Pratt SJ Ornitz DM Lung hypoplasia and neonatal death in Fgf9-null mice identify this gene as an essential regulator of lung mesenchyme Development 2001 128 2095 2106 11493531 10.1242/dev.128.11.2095

[B5] De Moerlooze L Spencer-Dene B Revest J Hajihosseini M Rosewell I Dickson C An important role for the IIIb isoform of fibroblast growth factor receptor 2 (FGFR2) in mesenchymal-epithelial signalling during mouse organogenesis Development 2000 127 483 492 10631169 10.1242/dev.127.3.483

[B6] Sekine K Ohuchi H Fujiwara M Yamasaki M Yoshizawa T Sato T Yagishita N Matsui D Koga Y Itoh N Kato S Fgf10 is essential for limb and lung formation Nat Genet 1999 21 138 141 10.1038/5096 9916808

[B7] Weinstein M Xu X Ohyama K Deng CX FGFR-3 and FGFR-4 function cooperatively to direct alveogenesis in the murine lung Development 1998 125 3615 3623 9716527 10.1242/dev.125.18.3615

[B8] Min H Danilenko DM Scully SA Bolon B Ring BD Tarpley JE DeRose M Simonet WS Fgf-10 is required for both limb and lung development and exhibits striking functional similarity to Drosophila branchless Genes Dev 1998 12 3156 3161 10.1101/gad.12.20.3156 9784490 PMC317210

[B9] Peters K Werner S Liao X Wert S Whitsett J Williams L Targeted expression of a dominant negative FGF receptor blocks branching morphogenesis and epithelial differentiation of the mouse lung EMBO J 1994 13 3296 3301 8045260 10.1002/j.1460-2075.1994.tb06631.x PMC395226

[B10] Usui H Shibayama M Ohbayashi N Konishi M Takada S Itoh N Fgf18 is required for embryonic lung alveolar development Biochem Biophys Res Commun 2004 322 887 892 10.1016/j.bbrc.2004.07.198 15336546

[B11] Ornitz DM Yayon A Flanagan JG Svahn CM Levi E Leder P Heparin is required for cell-free binding of basic fibroblast growth factor to a soluble receptor and for mitogenesis in whole cells Mol Cell Biol 1992 12 240 247 1309590 10.1128/mcb.12.1.240-247.1992 PMC364088

[B12] Yayon A Klagsbrun M Esko JD Leder P Ornitz DM Cell surface, heparin-like molecules are required for binding of basic fibroblast growth factor to its high affinity receptor Cell 1991 64 841 848 10.1016/0092-8674(91)90512-W 1847668

[B13] Rapraeger AC Krufka A Olwin BB Requirement of heparan sulfate for bFGF-mediated fibroblast growth and myoblast differentiation Science 1991 252 1705 1708 10.1126/science.1646484 1646484

[B14] Harding R Hooper SB Regulation of lung expansion and lung growth before birth J Appl Physiol 1996 81 209 224 8828667 10.1152/jappl.1996.81.1.209

[B15] Jesudason EC Exploiting mechanical stimuli to rescue growth of the hypoplastic lung Pediatr Surg Int 2007 23 827 836 10.1007/s00383-007-1956-0 17619196

[B16] Ai X Do AT Kusche-Gullberg M Lindahl U Lu K Emerson CP Jr Substrate specificity and domain functions of extracellular heparan sulfate 6-O-endosulfatases, QSulf1 and QSulf2 J Biol Chem 2006 281 4969 4976 16377625 10.1074/jbc.M511902200

[B17] Morimoto-Tomita M Uchimura K Werb Z Hemmerich S Rosen SD Cloning and characterization of two extracellular heparin-degrading endosulfatases in mice and humans J Biol Chem 2002 277 49175 49185 10.1074/jbc.M205131200 12368295 PMC2779716

[B18] Dhoot GK Gustafsson MK Ai X Sun W Standiford DM Emerson CP Jr Regulation of Wnt signaling and embryo patterning by an extracellular sulfatase Science 2001 293 1663 1666 10.1126/science.293.5535.1663 11533491

[B19] Kure S Yoshie O A syngeneic monoclonal antibody to murine Meth-A sarcoma (HepSS-1) recognizes heparan sulfate glycosaminoglycan (HS-GAG): cell density and transformation dependent alteration in cell surface HS-GAG defined by HepSS-1 J Immunol 1986 137 3900 3908 2431047

[B20] van den Born J van den Heuvel LP Bakker MA Veerkamp JH Assmann KJ Berden JH Production and characterization of a monoclonal antibody against human glomerular heparan sulfate Lab Invest 1991 65 287 297 1890809

[B21] David G Bai XM Van der Schueren B Cassiman JJ Van den Berghe H Developmental changes in heparan sulfate expression: in situ detection with mAbs J Cell Biol 1992 119 961 975 10.1083/jcb.119.4.961 1385449 PMC2289686

[B22] van Kuppevelt TH Dennissen MA van Venrooij WJ Hoet RM Veerkamp JH Generation and application of type-specific anti-heparan sulfate antibodies using phage display technology. Further evidence for heparan sulfate heterogeneity in the kidney J Biol Chem 1998 273 12960 12966 10.1074/jbc.273.21.12960 9582329

[B23] van Kuppevelt TH Jenniskens GJ Veerkamp JH ten Dam GB Dennissen MA Phage display technology to obtain antiheparan sulfate antibodies Methods Mol Biol 2001 171 519 534 11450265 10.1385/1-59259-209-0:519

[B24] Thompson SM Fernig DG Jesudason EC Losty PD van de Westerlo EM van Kuppevelt TH Turnbull JE Heparan sulphate phage display antibodies identify distinct epitopes with complex binding characteristics: insights into protein binding specificities J Biol Chem 2009 284 35621 35631 10.1074/jbc.M109.009712 19837661 PMC2790993

[B25] Thompson SM Connell MG Fernig DG Ten Dam GB van Kuppevelt TH Turnbull JE Jesudason EC Losty PD Novel 'phage display antibodies identify distinct heparan sulfate domains in developing mammalian lung Pediatr Surg Int 2007 10.1007/s00383-006-1864-8 17216534

[B26] Li JP Gong F Hagner-McWhirter A Forsberg E Abrink M Kisilevsky R Zhang X Lindahl U Targeted disruption of a murine glucuronyl C5-epimerase gene results in heparan sulfate lacking L-iduronic acid and in neonatal lethality J Biol Chem 2003 278 28363 28366 10.1074/jbc.C300219200 12788935

[B27] Ringvall M Ledin J Holmborn K van Kuppevelt T Ellin F Eriksson I Olofsson AM Kjellen L Forsberg E Defective heparan sulfate biosynthesis and neonatal lethality in mice lacking N-deacetylase/N-sulfotransferase-1 J Biol Chem 2000 275 25926 25930 10.1074/jbc.C000359200 10852901

[B28] Fan G Xiao L Cheng L Wang X Sun B Hu G Targeted disruption of NDST-1 gene leads to pulmonary hypoplasia and neonatal respiratory distress in mice FEBS Lett 2000 467 7 11 10.1016/S0014-5793(00)01111-X 10664446

[B29] Lin X Buff EM Perrimon N Michelson AM Heparan sulfate proteoglycans are essential for FGF receptor signaling during Drosophila embryonic development Development 1999 126 3715 3723 10433902 10.1242/dev.126.17.3715

[B30] Izvolsky KI Zhong L Wei L Yu Q Nugent MA Cardoso WV Heparan sulfates expressed in the distal lung are required for Fgf10 binding to the epithelium and for airway branching Am J Physiol 2003 285 L838 846 10.1152/ajplung.00081.2003 12818887

[B31] Izvolsky KI Shoykhet D Yang Y Yu Q Nugent MA Cardoso WV Heparan sulfate-FGF10 interactions during lung morphogenesis Dev Biol 2003 258 185 200 10.1016/S0012-1606(03)00114-3 12781692

[B32] Li M Shuman C Fei YL Cutiongco E Bender HA Stevens C Wilkins-Haug L Day-Salvatore D Yong SL Geraghty MT GPC3 mutation analysis in a spectrum of patients with overgrowth expands the phenotype of Simpson-Golabi-Behmel syndrome Am J Med Genet 2001 102 161 168 10.1002/1096-8628(20010801)102:2<161::AID-AJMG1453>3.0.CO;2-O 11477610

[B33] Neri G Gurrieri F Zanni G Lin A Clinical and molecular aspects of the Simpson-Golabi-Behmel syndrome Am J Med Genet 1998 79 279 283 10.1002/(SICI)1096-8628(19981002)79:4<279::AID-AJMG9>3.0.CO;2-H 9781908

[B34] Pilia G Hughes-Benzie RM MacKenzie A Baybayan P Chen EY Huber R Neri G Cao A Forabosco A Schlessinger D Mutations in GPC3, a glypican gene, cause the Simpson-Golabi-Behmel overgrowth syndrome Nat Genet 1996 12 241 247 10.1038/ng0396-241 8589713

[B35] Slavotinek AM Single gene disorders associated with congenital diaphragmatic hernia Am J Med Genet C Semin Med Genet 2007 145 172 183 10.1002/ajmg.c.30125 17436300

[B36] Costlow RD Manson JM The heart and diaphragm: target organs in the neonatal death induced by nitrofen (2,4-dichlorophenyl-p-nitrophenyl ether) Toxicology 1981 20 209 227 10.1016/0300-483X(81)90052-4 7256786

[B37] Kluth D Kangah R Reich P Tenbrinck R Tibboel D Lambrecht W Nitrofen-induced diaphragmatic hernias in rats: an animal model J Pediatr Surg 1990 25 850 854 10.1016/0022-3468(90)90190-K 2401939

[B38] Tenbrinck R Tibboel D Gaillard JL Kluth D Bos AP Lachmann B Molenaar JC Experimentally induced congenital diaphragmatic hernia in rats J Pediatr Surg 1990 25 426 429 10.1016/0022-3468(90)90386-N 2329458

[B39] Coleman C Zhao J Gupta M Buckley S Tefft JD Wuenschell CW Minoo P Anderson KD Warburton D Inhibition of vascular and epithelial differentiation in murine nitrofen-induced diaphragmatic hernia Am J Physiol 1998 274 L636 646 9575882 10.1152/ajplung.1998.274.4.L636

[B40] Tenbrinck R Gaillard JL Tibboel D Kluth D Lachmann B Molenaar JC Pulmonary vascular abnormalities in experimentally induced congenital diaphragmatic hernia in rats J Pediatr Surg 1992 27 862 865 10.1016/0022-3468(92)90385-K 1640335

[B41] Okoye BO Losty PD Lloyd DA Gosney JR Effect of prenatal glucocorticoids on pulmonary vascular muscularisation in nitrofen-induced congenital diaphragmatic hernia J Pediatr Surg 1998 33 76 80 10.1016/S0022-3468(98)90366-9 9473105

[B42] del Moral PM De Langhe SP Sala FG Veltmaat JM Tefft D Wang K Warburton D Bellusci S Differential role of FGF9 on epithelium and mesenchyme in mouse embryonic lung Dev Biol 2006 293 77 89 10.1016/j.ydbio.2006.01.020 16494859

[B43] Han RN Liu J Tanswell AK Post M Expression of basic fibroblast growth factor and receptor: immunolocalization studies in developing rat fetal lung Pediatr Res 1992 31 435 440 1318538 10.1203/00006450-199205000-00004

[B44] Lebeche D Malpel S Cardoso WV Fibroblast growth factor interactions in the developing lung Mech Dev 1999 86 125 136 10.1016/S0925-4773(99)00124-0 10446271

[B45] Smits NC Robbesom AA Versteeg EM van de Westerlo EM Dekhuijzen PN van Kuppevelt TH Heterogeneity of heparan sulfates in human lung Am J Respir Cell Mol Biol 2004 30 166 173 10.1165/rcmb.2003-0198OC 12896874

[B46] Rudland PS Platt-Higgins AM Wilkinson MC Fernig DG Immunocytochemical identification of basic fibroblast growth factor in the developing rat mammary gland: variations in location are dependent on glandular structure and differentiation J Histochem Cytochem 1993 41 887 898 10.1177/41.6.7686196 7686196

[B47] Jesudason EC Connell MG Fernig DG Lloyd DA Losty PD In vitro effects of growth factors on lung hypoplasia in a model of congenital diaphragmatic hernia J Pediatr Surg 2000 35 914 922 10.1053/jpsu.2000.6919 10873035

[B48] Jesudason EC Connell MG Fernig DG Lloyd DA Losty PD Heparin and in-vitro experimental lung hypoplasia Pediatr Surg Int 2000 16 247 251 10.1007/s003830050738 10898223

[B49] Jesudason EC Connell MG Fernig DG Lloyd DA Losty PD Early lung malformations in congenital diaphragmatic hernia J Pediatr Surg 2000 35 124 127 discussion 128 10.1016/S0022-3468(00)80028-7 10646789

[B50] Iritani I Experimental study on embryogenesis of congenital diaphragmatic hernia Anat Embryol 1984 169 133 139 10.1007/BF00303142 6742452

[B51] Keijzer R Liu J Deimling J Tibboel D Post M Dual-hit hypothesis explains pulmonary hypoplasia in the nitrofen model of congenital diaphragmatic hernia Am J Pathol 2000 156 1299 1306 10.1016/S0002-9440(10)65000-6 10751355 PMC1876880

[B52] Kamimura K Fujise M Villa F Izumi S Habuchi H Kimata K Nakato H Drosophila heparan sulfate 6-O-sulfotransferase (dHS6ST) gene. Structure, expression, and function in the formation of the tracheal system J Biol Chem 2001 276 17014 17021 10.1074/jbc.M011354200 11278892

[B53] Kamimura K Koyama T Habuchi H Ueda R Masu M Kimata K Nakato H Specific and flexible roles of heparan sulfate modifications in Drosophila FGF signaling J Cell Biol 2006 174 773 778 10.1083/jcb.200603129 16966419 PMC2064332

[B54] Habuchi H Nagai N Sugaya N Atsumi F Stevens RL Kimata K Mice deficient in heparan sulfate 6-O-sulfotransferase-1 exhibit defective heparan sulfate biosynthesis, abnormal placentation, and late embryonic lethality J Biol Chem 2007 282 15578 15588 10.1074/jbc.M607434200 17405882

[B55] Makarenkova HP Hoffman MP Beenken A Eliseenkova AV Meech R Tsau C Patel VN Lang RA Mohammadi M Differential interactions of FGFs with heparan sulfate control gradient formation and branching morphogenesis Sci Signal 2009 2 ra55 10.1126/scisignal.2000304 19755711 PMC2884999

[B56] Matsui R Brody JS Yu Q FGF-2 induces surfactant protein gene expression in foetal rat lung epithelial cells through a MAPK-independent pathway Cell Signal 1999 11 221 228 10.1016/S0898-6568(98)00070-9 10353697

[B57] Turnbull JE Fernig DG Ke Y Wilkinson MC Gallagher JT Identification of the basic fibroblast growth factor binding sequence in fibroblast heparan sulfate J Biol Chem 1992 267 10337 10341 1587820

[B58] Ashikari-Hada S Habuchi H Kariya Y Itoh N Reddi AH Kimata K Characterization of growth factor-binding structures in heparin/heparan sulfate using an octasaccharide library J Biol Chem 2004 279 12346 12354 14707131 10.1074/jbc.M313523200

[B59] Delehedde M Lyon M Gallagher JT Rudland PS Fernig DG Fibroblast growth factor-2 binds to small heparin-derived oligosaccharides and stimulates a sustained phosphorylation of p42/44 mitogen-activated protein kinase and proliferation of rat mammary fibroblasts Biochem J 2002 366 235 244 12000311 10.1042/BJ20011718 PMC1222755

[B60] Rahmoune H Chen HL Gallagher JT Rudland PS Fernig DG Interaction of heparan sulfate from mammary cells with acidic fibroblast growth factor (FGF) and basic FGF. Regulation of the activity of basic FGF by high and low affinity binding sites in heparan sulfate J Biol Chem 1998 273 7303 7310 10.1074/jbc.273.13.7303 9516424

[B61] White AC Lavine KJ Ornitz DM FGF9 and SHH regulate mesenchymal Vegfa expression and development of the pulmonary capillary network Development 2007 134 3743 3752 10.1242/dev.004879 17881491 PMC2099314

[B62] White AC Xu J Yin Y Smith C Schmid G Ornitz DM FGF9 and SHH signaling coordinate lung growth and development through regulation of distinct mesenchymal domains Development 2006 133 1507 1517 10.1242/dev.02313 16540513

[B63] Hecht HJ Adar R Hofmann B Bogin O Weich H Yayon A Structure of fibroblast growth factor 9 shows a symmetric dimer with unique receptor- and heparin-binding interfaces Acta Crystallogr D Biol Crystallogr 2001 57 378 384 10.1107/S0907444900020813 11223514

[B64] Plotnikov AN Eliseenkova AV Ibrahimi OA Shriver Z Sasisekharan R Lemmon MA Mohammadi M Crystal structure of fibroblast growth factor 9 reveals regions implicated in dimerization and autoinhibition J Biol Chem 2001 276 4322 4329 10.1074/jbc.M006502200 11060292

[B65] Weaver M Batts L Hogan BL Tissue interactions pattern the mesenchyme of the embryonic mouse lung Dev Biol 2003 258 169 184 10.1016/S0012-1606(03)00117-9 12781691

[B66] Rozario T DeSimone DW The extracellular matrix in development and morphogenesis: a dynamic view Dev Biol 2010 341 126 140 10.1016/j.ydbio.2009.10.026 19854168 PMC2854274

[B67] Gjorevski N Nelson CM Bidirectional extracellular matrix signaling during tissue morphogenesis Cytokine Growth Factor Rev 2009 20 459 465 10.1016/j.cytogfr.2009.10.013 19896886 PMC2787686

[B68] Wang N Tytell JD Ingber DE Mechanotransduction at a distance: mechanically coupling the extracellular matrix with the nucleus Nat Rev Mol Cell Biol 2009 10 75 82 19197334 10.1038/nrm2594

[B69] Schittny JC Miserocchi G Sparrow MP Spontaneous peristaltic airway contractions propel lung liquid through the bronchial tree of intact and fetal lung explants Am J Respir Cell Mol Biol 2000 23 11 18 10873148 10.1165/ajrcmb.23.1.3926

[B70] Jesudason EC Smith NP Connell MG Spiller DG White MR Fernig DG Losty PD Developing rat lung has a sided pacemaker region for morphogenesis-related airway peristalsis Am J Respir Cell Mol Biol 2005 32 118 127 15576668 10.1165/rcmb.2004-0304OC

[B71] Featherstone NC Jesudason EC Connell MG Fernig DG Wray S Losty PD Burdyga TV Spontaneous propagating calcium waves underpin airway peristalsis in embryonic rat lung Am J Respir Cell Mol Biol 2005 33 153 160 10.1165/rcmb.2005-0137OC 15891108

[B72] McCray PB Jr Spontaneous contractility of human fetal airway smooth muscle Am J Respir Cell Mol Biol 1993 8 573 580 8481238 10.1165/ajrcmb/8.5.573

[B73] Mailleux AA Kelly R Veltmaat JM De Langhe SP Zaffran S Thiery JP Bellusci S Fgf10 expression identifies parabronchial smooth muscle cell progenitors and is required for their entry into the smooth muscle cell lineage Development 2005 132 2157 2166 10.1242/dev.01795 15800000

[B74] Acosta JM Thebaud B Castillo C Mailleux A Tefft D Wuenschell C Anderson KD Bourbon J Thiery JP Bellusci S Warburton D Novel mechanisms in murine nitrofen-induced pulmonary hypoplasia: FGF-10 rescue in culture Am J Physiol 2001 281 L250 257 10.1152/ajplung.2001.281.1.L250 11404268

[B75] Jesudason EC Smith NP Connell MG Spiller DG White MR Fernig DG Losty PD Peristalsis of airway smooth muscle is developmentally regulated and uncoupled from hypoplastic lung growth Am J Physiol 2006 291 L559 565 10.1152/ajplung.00498.2005 16603591

[B76] Featherstone NC Connell MG Fernig DG Wray S Burdyga TV Losty PD Jesudason EC Airway smooth muscle dysfunction precedes teratogenic congenital diaphragmatic hernia and may contribute to hypoplastic lung morphogenesis Am J Respir Cell Mol Biol 2006 35 571 578 10.1165/rcmb.2006-0079OC 16728706

[B77] LeBleu VS Macdonald B Kalluri R Structure and function of basement membranes Exp Biol Med 2007 232 1121 1129 10.3181/0703-MR-72 17895520

[B78] Murdoch AD Liu B Schwarting R Tuan RS Iozzo RV Widespread expression of perlecan proteoglycan in basement membranes and extracellular matrices of human tissues as detected by a novel monoclonal antibody against domain III and by in situ hybridization J Histochem Cytochem 1994 42 239 249 10.1177/42.2.7507142 7507142

[B79] Groffen AJ Ruegg MA Dijkman H van de Velden TJ Buskens CA van den Born J Assmann KJ Monnens LA Veerkamp JH van den Heuvel LP Agrin is a major heparan sulfate proteoglycan in the human glomerular basement membrane J Histochem Cytochem 1998 46 19 27 10.1177/002215549804600104 9405491

[B80] Groffen AJ Buskens CA van Kuppevelt TH Veerkamp JH Monnens LA van den Heuvel LP Primary structure and high expression of human agrin in basement membranes of adult lung and kidney Eur J Biochem 1998 254 123 128 10.1046/j.1432-1327.1998.2540123.x 9652404

[B81] Miosge N Simniok T Sprysch P Herken R The collagen type XVIII endostatin domain is co-localized with perlecan in basement membranes in vivo J Histochem Cytochem 2003 51 285 296 10.1177/002215540305100303 12588956

[B82] Halfter W Dong S Schurer B Cole GJ Collagen XVIII is a basement membrane heparan sulfate proteoglycan J Biol Chem 1998 273 25404 25412 10.1074/jbc.273.39.25404 9738008

[B83] Brauer PR Keller KM Keller JM Concurrent reduction in the sulfation of heparan sulfate and basement membrane assembly in a cell model system Development 1990 110 805 813 2088721 10.1242/dev.110.3.805

[B84] Yurchenco PD Cheng YS Schittny JC Heparin modulation of laminin polymerization J Biol Chem 1990 265 3981 3991 2303489

[B85] Tsilibary EC Koliakos GG Charonis AS Vogel AM Reger LA Furcht LT Heparin type IV collagen interactions: equilibrium binding and inhibition of type IV collagen self-assembly J Biol Chem 1988 263 19112 19118 3198614

[B86] Dowd CJ Cooney CL Nugent MA Heparan sulfate mediates bFGF transport through basement membrane by diffusion with rapid reversible binding J Biol Chem 1999 274 5236 5244 10.1074/jbc.274.8.5236 9988774

[B87] Friedl A Chang Z Tierney A Rapraeger AC Differential binding of fibroblast growth factor-2 and -7 to basement membrane heparan sulfate: comparison of normal and abnormal human tissues Am J Pathol 1997 150 1443 1455 9094999 PMC1858159

[B88] Vlodavsky I Fuks Z Ishai-Michaeli R Bashkin P Levi E Korner G Bar-Shavit R Klagsbrun M Extracellular matrix-resident basic fibroblast growth factor: implication for the control of angiogenesis J Cell Biochem 1991 45 167 176 10.1002/jcb.240450208 1711529

[B89] Folkman J Klagsbrun M Sasse J Wadzinski M Ingber D Vlodavsky I A heparin-binding angiogenic protein--basic fibroblast growth factor--is stored within basement membrane Am J Pathol 1988 130 393 400 3277442 PMC1880518

[B90] Willem M Miosge N Halfter W Smyth N Jannetti I Burghart E Timpl R Mayer U Specific ablation of the nidogen-binding site in the laminin gamma1 chain interferes with kidney and lung development Development 2002 129 2711 2722 12015298 10.1242/dev.129.11.2711

[B91] Bader BL Smyth N Nedbal S Miosge N Baranowsky A Mokkapati S Murshed M Nischt R Compound genetic ablation of nidogen 1 and 2 causes basement membrane defects and perinatal lethality in mice Mol Cell Biol 2005 25 6846 6856 10.1128/MCB.25.15.6846-6856.2005 16024816 PMC1190363

[B92] Lafont J Baroukh B Berdal A Colombier ML Barritault D Caruelle JP Saffar JL RGTA11, a new healing agent, triggers developmental events during healing of craniotomy defects in adult rats Growth factors 1998 16 23 38 10.3109/08977199809017489 9777368

[B93] Meddahi A Alexakis C Papy D Caruelle JP Barritault D Heparin-like polymer improved healing of gastric and colic ulceration J Biomed Mater Res 2002 60 497 501 10.1002/jbm.1293 11920675

[B94] Garcia-Filipe S Barbier-Chassefiere V Alexakis C Huet E Ledoux D Kerros ME Petit E Barritault D Caruelle JP Kern P RGTA OTR4120, a heparan sulfate mimetic, is a possible long-term active agent to heal burned skin J Biomed Mater Res A 2007 80 75 84 16958049 10.1002/jbm.a.30874

[B95] Tong M Zbinden MM Hekking IJ Vermeij M Barritault D van Neck JW RGTA OTR 4120, a heparan sulfate proteoglycan mimetic, increases wound breaking strength and vasodilatory capability in healing rat full-thickness excisional wounds Wound Repair Regen 2008 16 294 299 10.1111/j.1524-475X.2008.00368.x 18318813

[B96] Zakine G Barbier V Garcia-Filipe S Luboinski J Papy-Garcia D Chachques JC Carpentier A Barritault D Matrix therapy with RGTA OTR4120 improves healing time and quality in hairless rats with deep second-degree burns Plast Reconstr Surg 2011 127 541 550 10.1097/PRS.0b013e318200a910 21285759

[B97] Griffiths GO Burns S Noble SI Macbeth FR Cohen D Maughan TS FRAGMATIC: a randomised phase III clinical trial investigating the effect of fragmin added to standard therapy in patients with lung cancer BMC Cancer 2009 9 355 10.1186/1471-2407-9-355 19807917 PMC2761945

[B98] Lewis KD Robinson WA Millward MJ Powell A Price TJ Thomson DB Walpole ET Haydon AM Creese BR Roberts KL A phase II study of the heparanase inhibitor PI-88 in patients with advanced melanoma Invest New Drugs 2008 26 89 94 10.1007/s10637-007-9080-5 17891338

[B99] Basche M Gustafson DL Holden SN O'Bryant CL Gore L Witta S Schultz MK Morrow M Levin A Creese BR A phase I biological and pharmacologic study of the heparanase inhibitor PI-88 in patients with advanced solid tumors Clin Cancer Res 2006 12 5471 5480 10.1158/1078-0432.CCR-05-2423 17000682

[B100] Patel VN Likar KM Zisman-Rozen S Cowherd SN Lassiter KS Sher I Gallagher JT Yates EA Turnbull JE Ron D Hoffman MP Specific heparan sulfate structures modulate FGF10-mediated submandibular gland epithelial morphogenesis and differentiation J Biol Chem 2008 10.1074/jbc.M709995200 PMC2431040 18230614

[B101] Patey SJ Edwards EA Yates EA Turnbull JE Heparin derivatives as inhibitors of BACE-1, the Alzheimer's beta-secretase, with reduced activity against factor Xa and other proteases J Med Chem 2006 49 6129 6132 10.1021/jm051221o 17004727

[B102] Duchesne L Gentili D Comes-Franchini M Fernig DG Robust ligand shells for biological applications of gold nanoparticles Langmuir 2008 24 13572 13580 10.1021/la802876u 18991409

[B103] Studier FW Protein production by auto-induction in high density shaking cultures Protein Expr Purif 2005 41 207 234 10.1016/j.pep.2005.01.016 15915565

