# Supplementary figures and images for: Structure and epitope distribution of heparan sulfate is disrupted in experimental lung hypoplasia: a glycobiological epigenetic cause for malformation?
Source: BMC Dev Biol. 2011 Jun 14;11:38. doi: 10.1186/1471-213X-11-38 (PMC3127989; doi:10.1186/1471-213X-11-38)

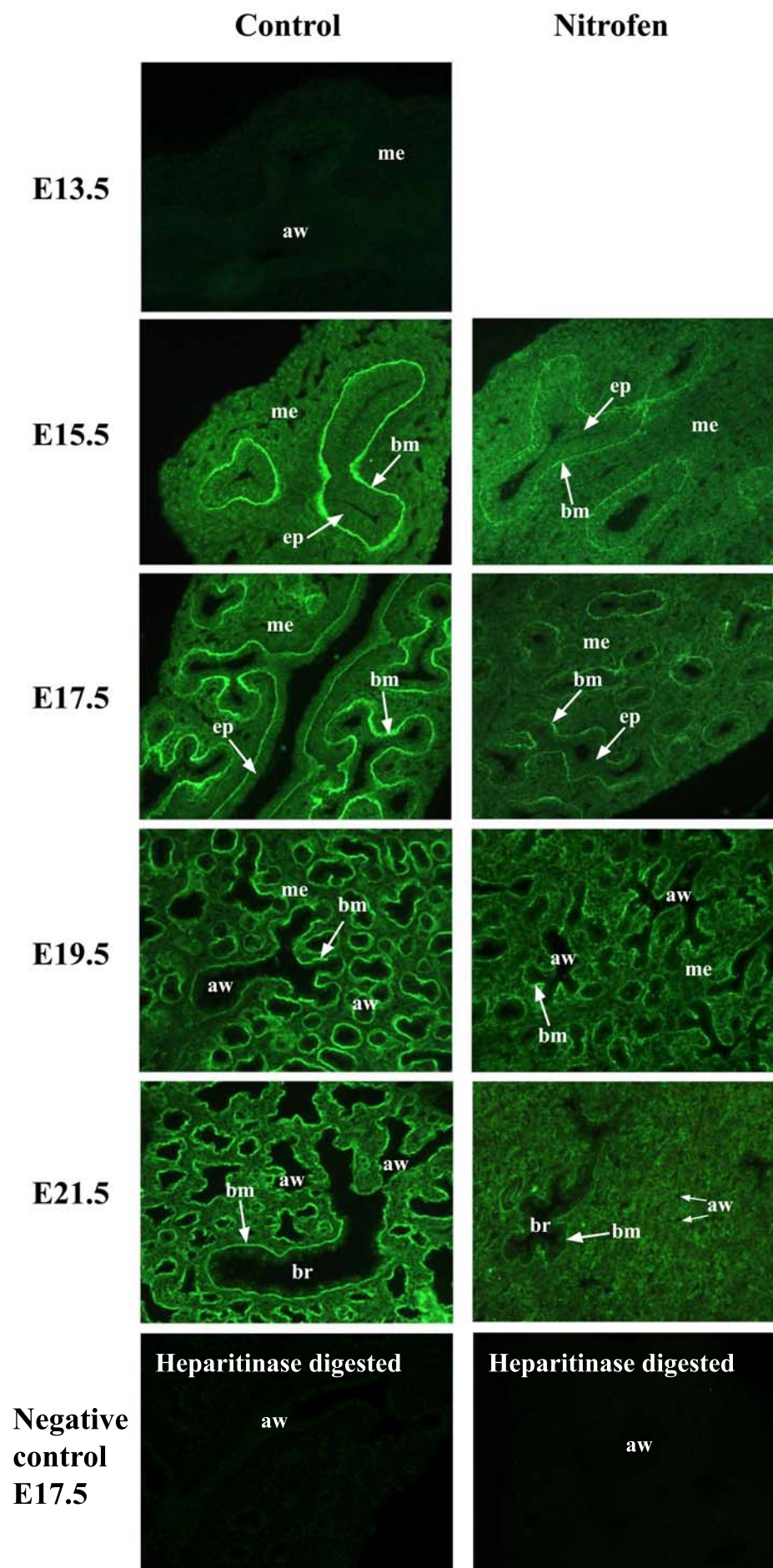

Supplement: Additional file 1 — HS3B7V. Immunohistochemical staining of E13.5 - E21.5 normal lungs and E15.5 - E21.5 hypoplastic lungs with HS3B7V. The HS3B7V HS epitope is localised to the epithelial basement membrane in both control and hypoplastic lungs from E15.5. However, in hypoplastic lungs, expression of this HS structure is reduced and staining of epithelial basement membranes is irregular. In addition, in nitrofen E19.5 lungs, there is additional weak staining identified in sub-epithelial mesenchyme. As a negative control, endogenous HS was digested with heparitinase prior to antibody incubation. (aw) airway, (mes) mesenchyme, (ep) epithelium, (bm) basement membrane, (br) bronchus. [file 1471-213X-11-38-S1.PDF]

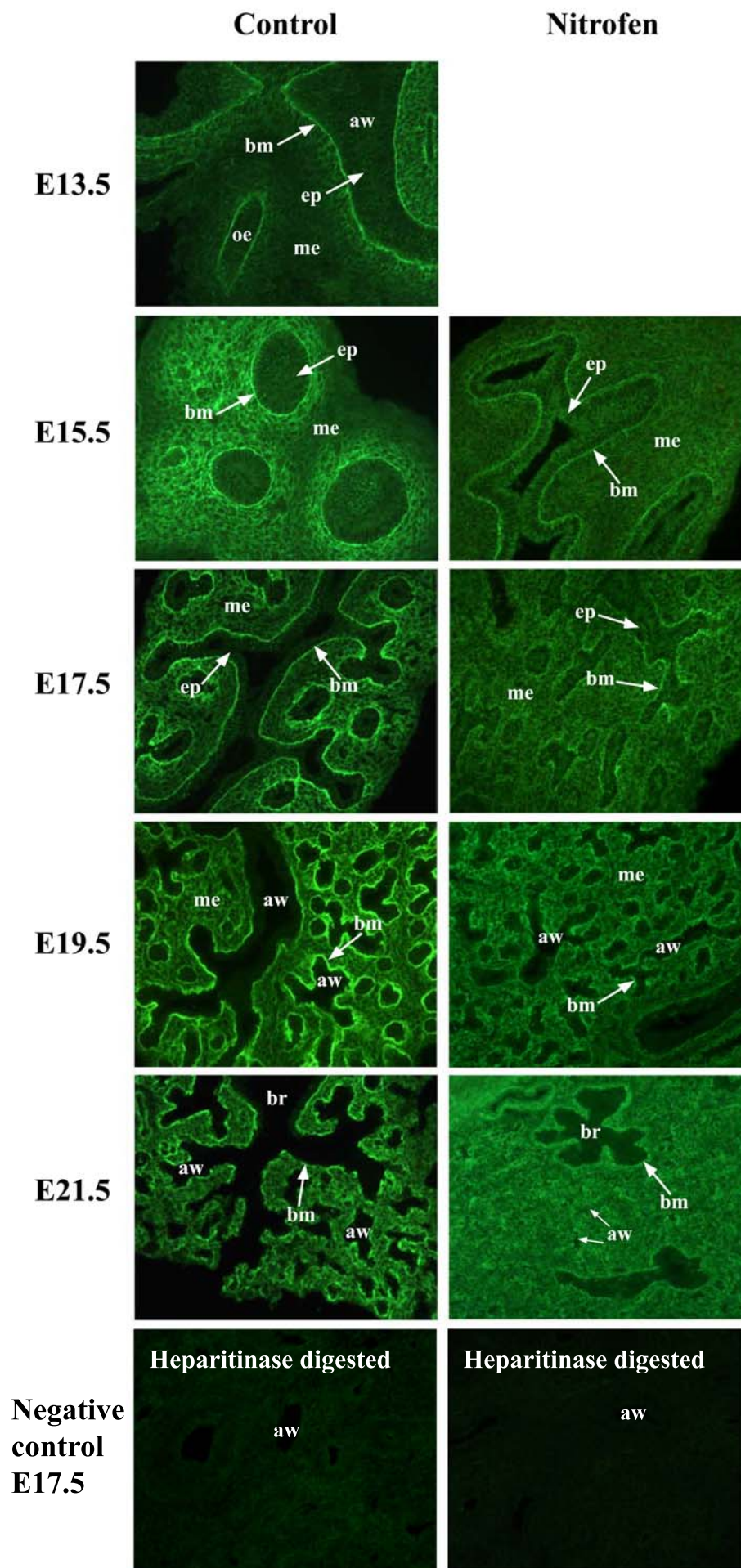

Supplement: Additional file 2 — HS4E4V. Immunohistochemical staining of E13.5 - E21.5 normal lungs and E15.5 - E21.5 hypoplastic lungs with HS4E4V. In normal lungs, the HS4E4V HS epitope is present in epithelial basement membranes and the surrounding mesenchyme, particularly in sub-epithelial areas adjacent to distal airways. In hypoplastic lungs, expression of this epitope is severely reduced, particularly in epithelial basement membranes and mesenchyme of E15.5 and E17.5 lungs. As a negative control, endogenous HS was digested with heparitinase prior to antibody incubation. (aw) airway, (oe) oesophagus, (mes) mesenchyme, (ep) epithelium, (bm) basement membrane, (br) bronchus. [file 1471-213X-11-38-S2.PDF]

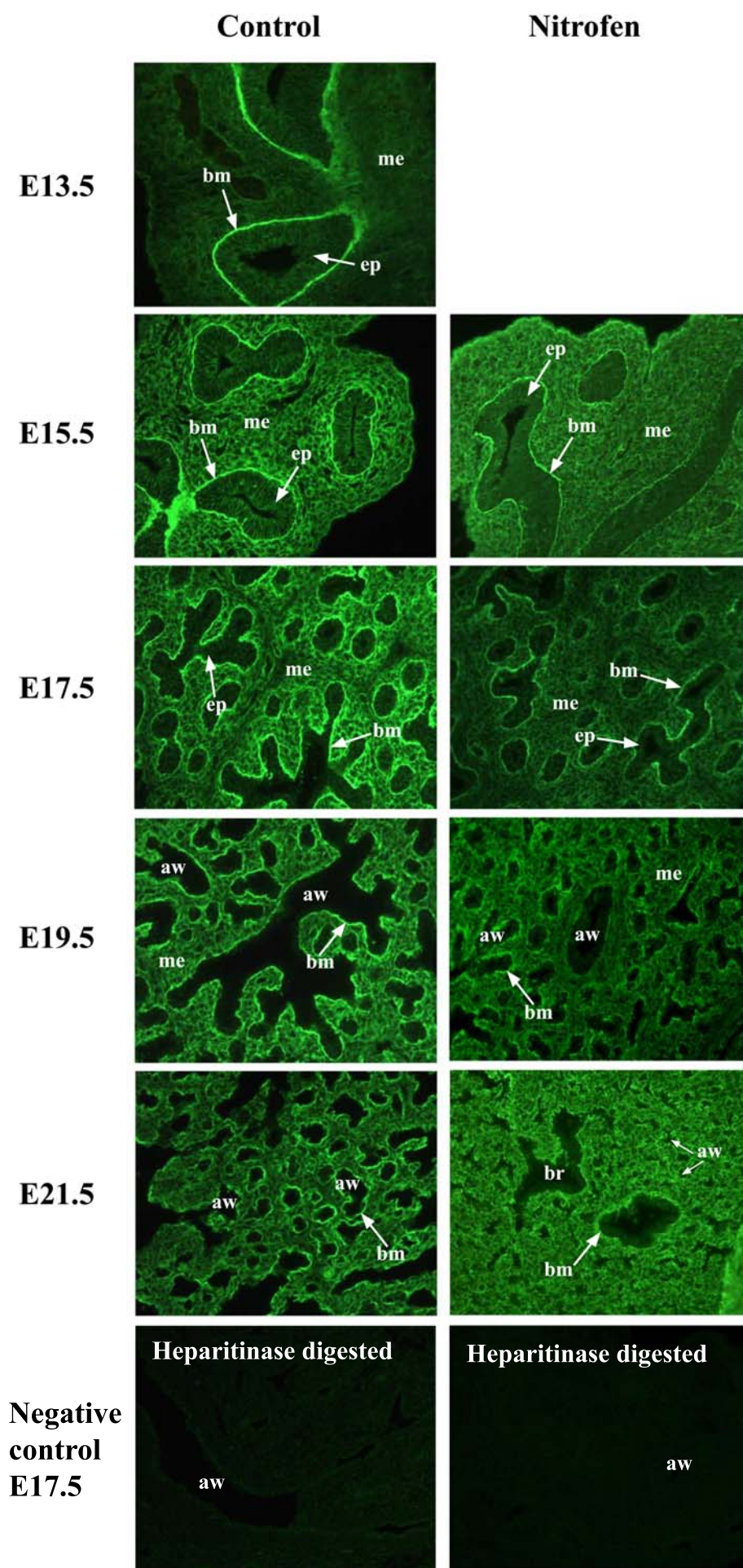

Supplement: Additional file 3 — HS3A8V. Immunohistochemical staining of E13.5 - E21.5 normal lungs and E15.5 - E21.5 hypoplastic lungs with HS3A8V. In normal lungs, the HS epitope recognised by HS3A8V is restricted to epithelial basement membranes at E13.5. From E15.5, distribution of the epitope is more widespread and is present in epithelial basement membranes and throughout the mesenchyme, particularly in sub-epithelial mesenchyme. Epithelial cells also display this HS structure transiently at E15.5 and (more weakly) at E17.5. In hypoplastic lungs, mesenchymal expression of the HS3A8V epitope is reduced, particularly at E15.5 and E17.5, and epithelial staining observed in normal lungs is lost. Additionally, irregularities in epithelial basement membrane staining are observed. As a negative control, endogenous HS was digested with heparitinase prior to antibody incubation. (aw) airway, (mes) mesenchyme, (ep) epithelium, (bm) basement membrane, (br) bronchus. [file 1471-213X-11-38-S3.PDF]

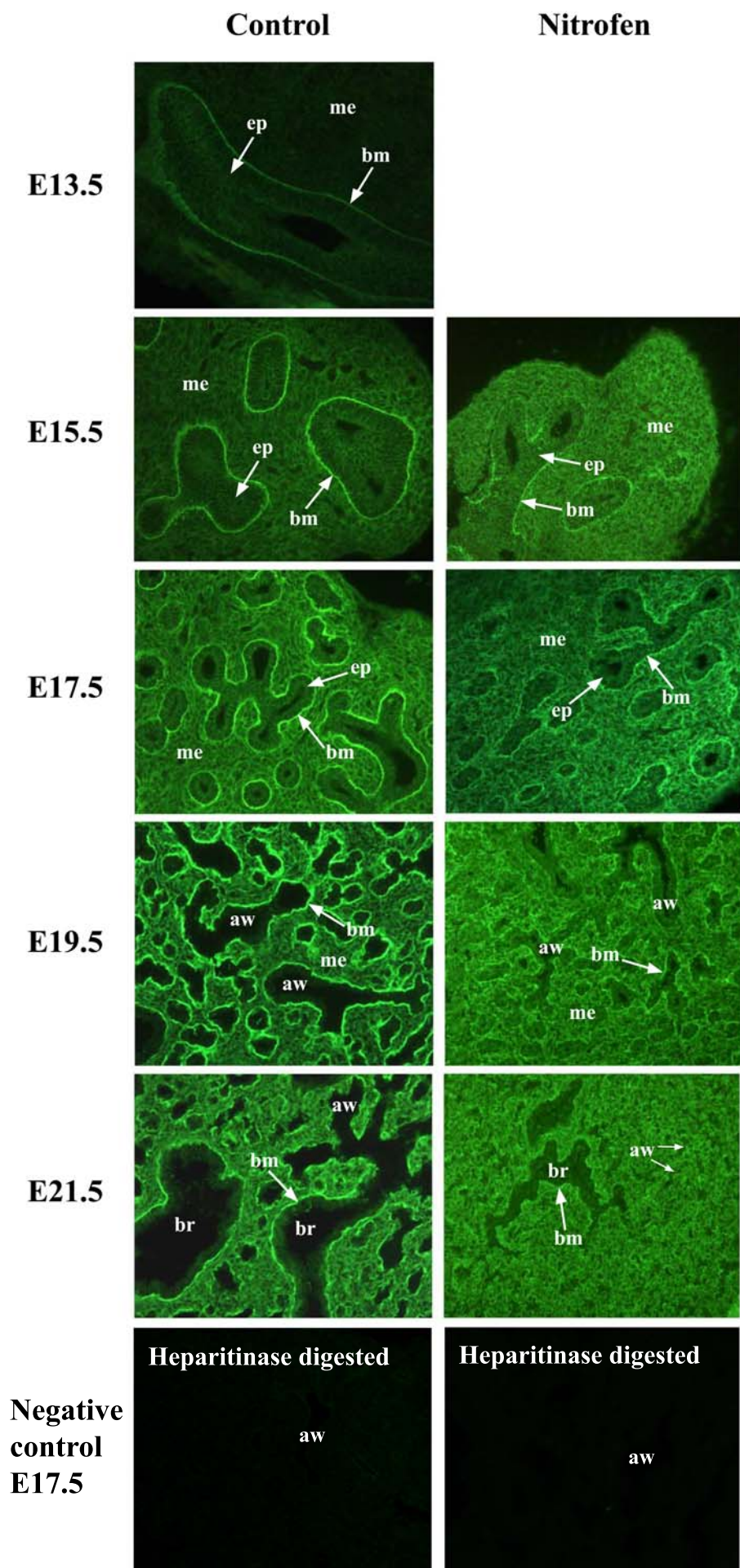

Supplement: Additional file 4 — AO4B08V. Immunohistochemical staining of E13.5 - E21.5 normal lungs and E15.5 - E21.5 hypoplastic lungs with AO4B08V. Expression of the AO4B08V HS epitope increases during the course of normal lung development. At E13.5, it is only weakly expressed by epithelial basement membranes, and at E15.5, is additionally displayed at a low level in the mesenchyme and airway epithelium. From E17.5 - E21.5, levels of this epitope increases in basement membranes and throughout the mesenchyme. In hypoplastic lungs, however, expression of the AO4B08V epitope is reduced in the epithelium and underlying basement membranes, and in addition, basement membranes appear discontinuous. In lung mesenchyme, however, the AO4B08V epitope structure is displayed at a higher level compared to normal lungs. As a negative control, endogenous HS was digested with heparitinase prior to antibody incubation. (aw) airway, (mes) mesenchyme, (ep) epithelium, (bm) basement membrane, (br) bronchus. [file 1471-213X-11-38-S4.PDF]

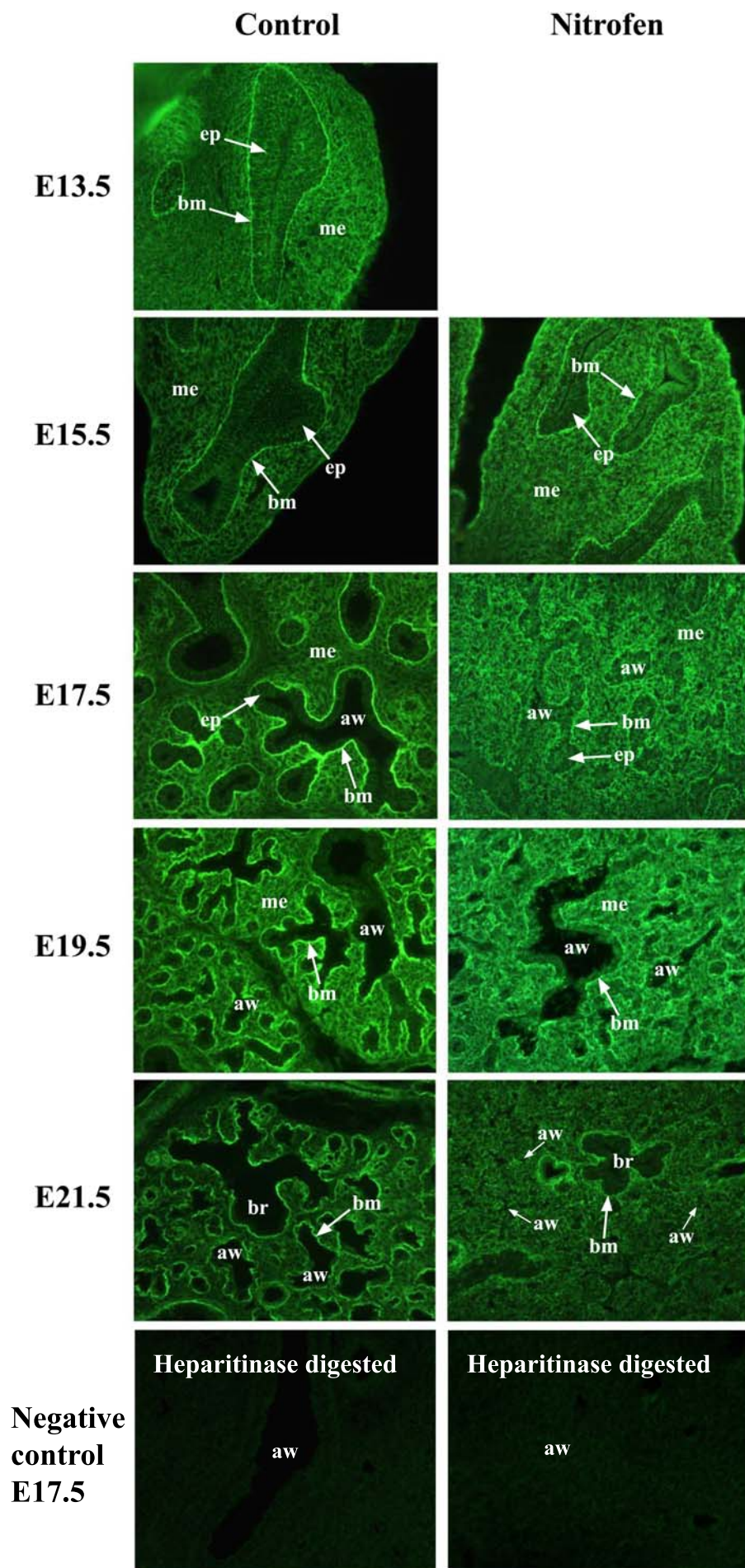

Supplement: Additional file 5 — EV3C3V. Immunohistochemical staining of E13.5 - E21.5 normal lungs and E15.5 - E21.5 hypoplastic lungs with EV3C3V. In normal lungs, the EV3C3V epitope is displayed by the epithelium at E13.5 - E17.5 and in the underlying basement membranes at E13.5 - E21.5. A gradient of epitope expression is observed in the mesenchyme, with highest levels in sub-epithelial mesenchyme around smaller, distal airways and lower levels in sub-mesothelial mesenchyme. However, in hypoplastic lungs, this gradient of mesenchymal expression is lost, and the EV3C3V epitope is more extensively and evenly distributed throughout the entire mesenchyme. In addition, epithelial staining is lost from hypoplastic lungs and basement membrane staining is irregular. As a negative control, endogenous HS was digested with heparitinase prior to antibody incubation. (aw) airway, (mes) mesenchyme, (ep) epithelium, (bm) basement membrane, (br) bronchus. [file 1471-213X-11-38-S5.PDF]

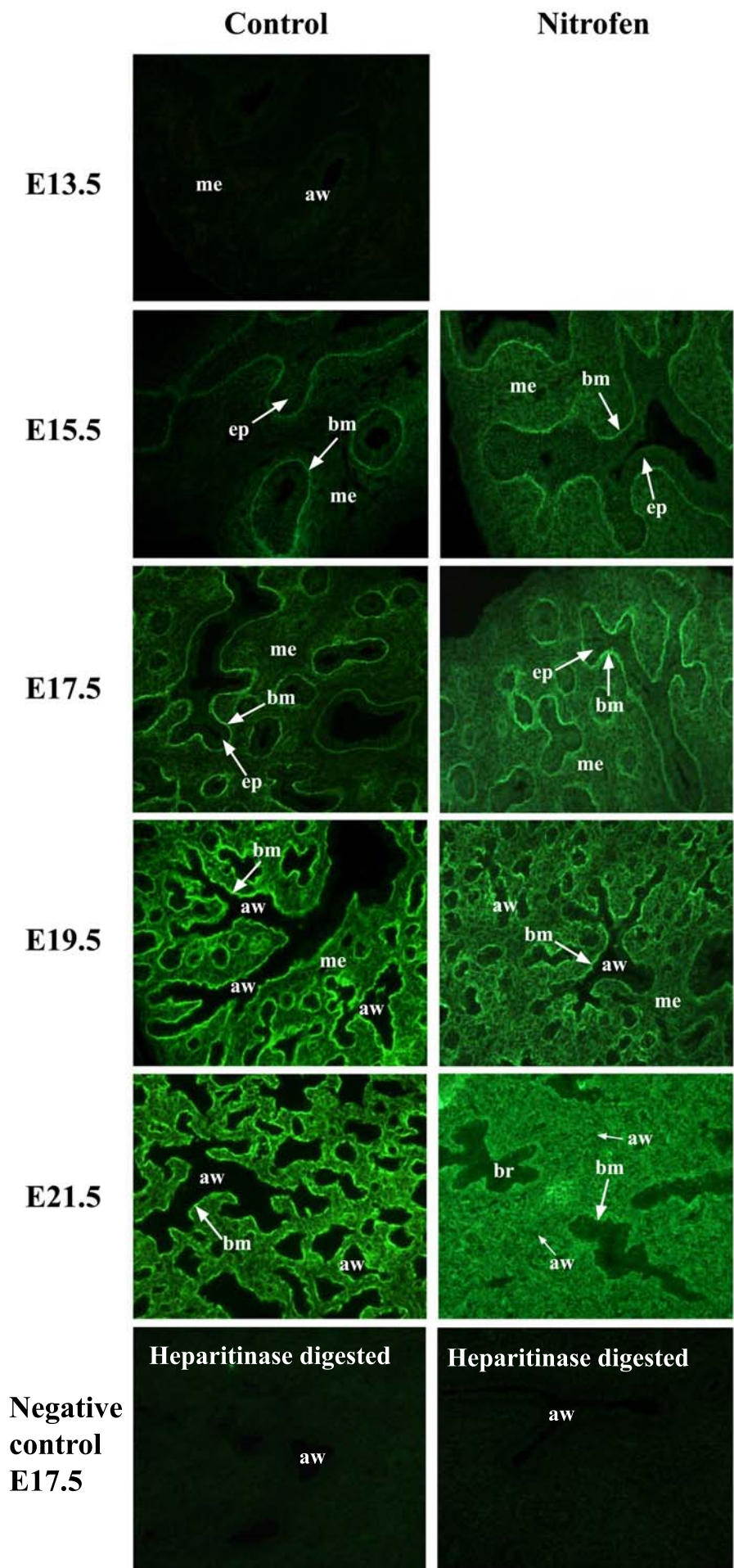

Supplement: Additional file 6 — EW4G1V. Immunohistochemical staining of E13.5 - E21.5 normal lungs and E15.5 - E21.5 hypoplastic lungs with EW4G1V. In normal developing lungs, the HS structure identified by EW4G1V is absent at E13.5. From E15.5 onwards, however, it is present in all epithelial basement membranes and also at a low level in the mesenchyme, with increased levels at E21.5. This epitope is transiently expressed by the epithelium at E15.5. In hypoplastic lungs, levels of this epitope appear to be raised somewhat in the mesenchyme compared to normal lungs and simultaneously reduced in epithelial basement membranes. As a negative control, endogenous HS was digested with heparitinase prior to antibody incubation. (aw) airway, (mes) mesenchyme, (ep) epithelium, (bm) basement membrane, (br) bronchus. [file 1471-213X-11-38-S6.PDF]
